# Switchable Polymerization Catalysis Using a Tin(II) Catalyst and Commercial Monomers to Toughen Poly(l-lactide)

**DOI:** 10.1021/acsmacrolett.1c00216

**Published:** 2021-06-08

**Authors:** Nattawut Yuntawattana, Georgina L. Gregory, Leticia Peña Carrodeguas, Charlotte K. Williams

**Affiliations:** Chemistry Research Laboratory, Department of Chemistry, University of Oxford, 12 Mansfield Road, Oxford OX1 3TA, U.K.

## Abstract

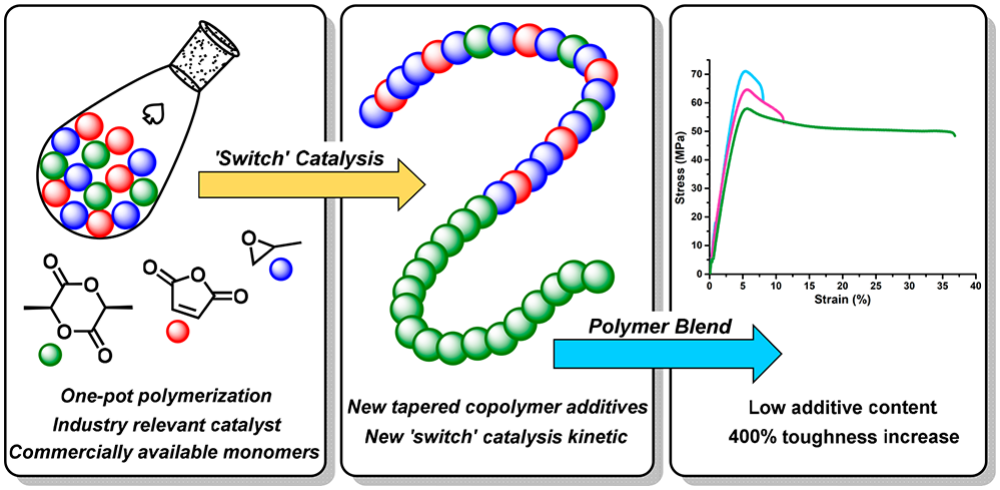

Sustainable plastics sourced without virgin petrochemicals, that are easily recyclable and with potential for degradation at end of life, are urgently needed. Here, copolymersand blends meeting these criteria are efficiently prepared using a single catalyst and existing commercial monomers l-lactide, propylene oxide, and maleic anhydride. The selective, one-reactor polymerization applies an industry-relevant tin(II) catalyst. Tapered, miscible block polyesters are formed with alkene groups which are postfunctionalized to modulate the polymer glass transition temperature. The polymers are blended at desirable low weight fractions (2 wt %) with commercial poly(l-lactide) (PLLA), increasing toughness, and elongation at break without compromising the elastic modulus, tensile strength, or thermal properties. The selective polymerization catalysis, using commercial monomers and catalyst, provides a straightforward means to improve bioplastics performances.

Poly(l-lactide) (PLLA) is the largest scale commercial sustainable plastic: it is sourced from sugar, can be recycled, and is industrially compostable.^1−7^ One limitation is its brittleness which requires 10–20 wt % of additives to formulate useful products; there is increasing concern about the influences of such additives during recycling or if leached into the environment.^8−13^ Small molecule additives become mobile over time, compromising mechanical performances, and may leach when PLLA is submerged in liquids.^14,15^ Blending PLLA with commercial elastomeric polymers improves elongation at break but often at the expense of Young’s modulus and tensile strength; this approach also usually necessitates a compatibilizer.^16^ Modifying lactide to introduce plasticizing substituents has been attempted, but preparing functionalized lactides requires multistep, hard-to-scale syntheses. Most substituted lactides show compromised polymerization thermodynamics.^17^ PLLA-containing copolyesters are needed, and to accelerate implementation any new methods to make them must be compatible with existing PLLA manufacturing. New monomers should be avoided, or rather innovation using the existing scalable monomer palette should be prioritized.

PLLA is industrially produced by controlled l-lactide ring-opening polymerization (ROP),^7^ catalyzed by Sn(II) alkoxides generated *in situ* from Sn(II) precursors and alcohols.^18,19^ Polyesters are also controllably prepared by epoxide and anhydride ring-opening copolymerization (ROCOP) which is thermodynamically feasible for many functional monomers.^20,21^ Importantly, such monomers are common in current polymer manufacturing and have acceptable costs. Efficient epoxide/anhydride ROCOP catalysts are known,^22−27^ and relevant here, Phomphrai and co-workers recently reported a tin(II) catalyst for cyclohexene oxide/phthalic anhydride ROCOP yielding a poly(ester-*ran*-ether) ABB structure.^28^ Switchable polymerization catalysis enchains mixtures of epoxides, anhydrides, and lactones using one catalyst in a single reactor process.^4,29−39^ It is applicable to many monomers, organometallic, inorganic, or organocatalysts,^40−43^ but until now, commercially relevant tin(II) catalysts were unexplored. Here, a Sn(II) alkoxide catalyst for switchable polymerization catalysis is investigated using only monomers which are both commercial and produced at large scale: l-lactide (l-LA = 1.8 Mt/annum), propylene oxide (PO = 9 Mt/annum), and maleic anhydride (MA = 3 Mt/annum).^44−46^

Before investigating the mixed monomer reactions, the tin(II) catalyst was tested for the two constituent polymerizations: PO/MA ROCOP and LA ROP. The catalyst was prepared *in situ* from tin(II) bis(methoxide) and two equivalents of benzyl alcohol (Table S1). The ring-opening copolymerization of propylene oxide and maleic anhydride, conducted using [Sn(OMe)_2_]:[BnOH]:[MA]:[PO] = 1:2:100:1000, at 45 °C, showed complete anhydride consumption, within 5 h, yielding a poly(ester-*ran*-ether) (PE). The polymer comprises ∼67–69% ether linkages and maintains this composition as the reaction progresses; its structure is consistent with an average ratio of 1:2 for ester:ether linkages (Figures S1–S4). PO/MA ROCOP is a challenging monomer set, and compared with other reported catalysts, the tin(II) alkoxide species shows significantly faster rates and much higher MA conversions (Table S2 and Figure S5). The same catalyst system also shows good performance in l-LA ROP, under equivalent conditions ([Sn(OMe)_2_]:[BnOH]:[l-LA]:[PO] = 1:2:100:1000, 45 °C), reaching quantitative monomer conversion within 3 h. Although excess propylene oxide was present, it remained unreacted, and only PLLA formed. In terms of thermal properties, PE is amorphous with a *T*_g_ = −19 °C, while PLLA is semi-crystalline, with *T*_m_ = 159 °C (Figures S6–S9).

Given the promising polymerization performances, switchable catalysis using mixtures was investigated ([Sn(OMe)_2_]:[BnOH]:[MA]:[l-LA]:[PO] = 1:2:100:100:1000; [Sn(OMe)_2_] = 14.3 mM, 45 °C). Aliquot analysis indicated the formation of a tapered block polyester, **P1**, where maleic anhydride/propylene oxide ROCOP occurs first, followed by a random enchainment and completed by a PLLA block (Table S3, Figures 1, 2, and S10–S16). To test for copolymer formation, rather than polymer mixtures, the ^1^H NMR spectra of crude **P1** and a purified sample were compared: both showed the same compositions (Figure S10). Further, the crude polymer samples show continual and predictable molar mass increases with monomer conversions and narrow dispersity throughout the polymerization (*Đ* ≤ 1.37) (Figure 1). The ^1^H DOSY NMR spectrum shows a single diffusion coefficient, whereas a blend of PE and PLLA shows two different diffusion coefficients (Figures S15 and S16). **P1** is amorphous with a single *T*_g_ (−5 °C), consistent with the two blocks being fully miscible (Figure S17). It shows a two-step degradation profile (238 °C = PLLA; 298 °C = PE block) (Figure S18). Aliquot analyses, using ^1^H NMR spectroscopy, determined monomer concentration–time relationships (Figure S19). The ring-opening copolymerization (PO/MA) occurs with a zeroth-order dependence on MA, *k*_obs_ = 23.1 ± 0.5 M h^–1^. Such kinetics are consistent with fast anhydride insertion into the tin alkoxide intermediate in the catalytic cycle. The lactide ring-opening polymerization stage shows a first-order dependence on lactide, *k*_obs_ = 2.74 ± 0.03 × 10^–6^ h^–1^. Polymerizations in NMR tubes also confirm the formation of a tapered block polyester (Figure 2 and Schemes S1 and S2). The three-stage reaction involves: (1) PO/MA ROCOP, until ∼50% anhydride consumption (Stage I). (2) Simultaneous l-LA ROP and PO/MA ROCOP, until complete anhydride consumption (l-LA = 50%) (Stage II). (3) l-LA ROP until complete lactide consumption (Stage III). The data suggest that maleic anhydride insertion occurs significantly faster than l-LA – such behavior is fully consistent with previous switch catalysts and rationalizes the initial period of selective ROCOP (PO/MA).^32,34−36,40^ In contrast to most switch catalysts, though, after ∼50% anhydride conversion l-LA is also simultaneously enchained. Prior DFT and kinetics investigations of switch catalysts show that monomer enchainment is usually determined by both the transition state energy and linkage stabilities.^37^ These prior investigations applied lactones that formed alkoxide linkages that were significantly less stable than the carboxylate linkages formed from anhydride insertion.^37^ Tin(II) lactide polymerization catalysts are known to form stable five-membered stannocycle intermediates.^47,48^ Such stannocycles should significantly enhance the alkoxide linkage stability, after lactide insertion, and may rationalize the parallel insertion of lactide and maleic anhydride. Once the anhydride is consumed, epoxide ring opening ceases, and the remaining l-LA is polymerized to PLLA.

**Figure 1:**
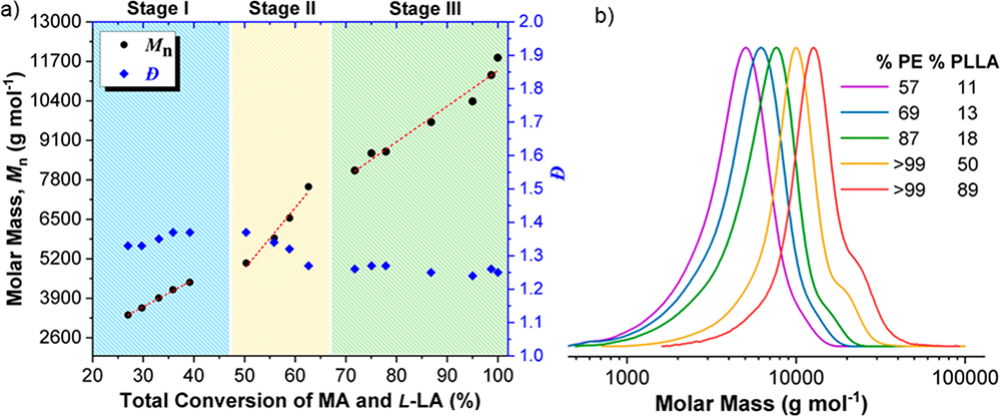
(a) Plot of **P1** molar mass (*M*_n_) and dispersity (*Đ*) vs total monomer conversion. (b) GPC stack plot for aliquots removed during **P1** formation (NB: the small “shoulder” at high PE conversion likely arises from minor maleate cross-linking reactions).

**Figure 2:**
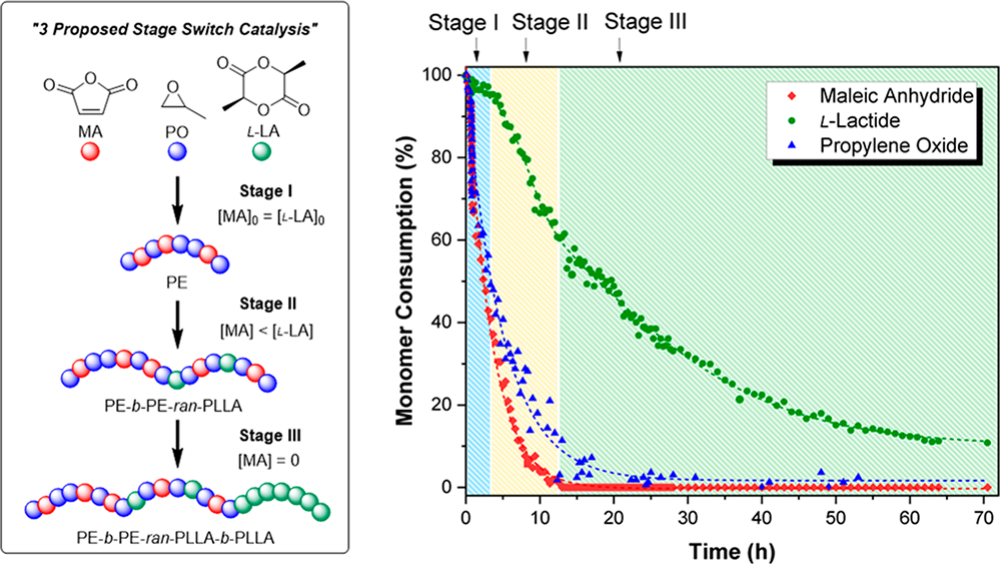
Switchable polymerization using PO/MA/l-LA with conversion *vs* time data to illustrate the reaction stages. The polymer structures (chem-draws) are shown in Scheme S2.

**P1** features miscible PLLA and alkene-functionalized polymer blocks as evidenced by its single glass transition temperature and suppressed PLLA block crystallinity (Figure S17). It is, therefore, expected to be miscible with bulk PLLA and may be suitable to toughen it.^11^ As a proof of concept, a series of films were prepared from homogeneous chloroform solutions of **P1** (2–20 wt %) and PLLA (*M*_n_ = 54 600 g mol^–1^, *Đ* = 1.73) cast into a PTFE mold and dried (fume cupboard, 25 °C, 20 h; vacuum oven, 60 °C, 72–96 h) until no solvent residues were detected (Figure S20). Dumbbell-shaped specimens, cut according to ISO 572-2 type 5B, were used for tensile mechanical investigations (Tables 1, 2, S4, and S5 and Figures 3, 4, and S21–S24). Compared to control PLLA samples, adding just 2 wt % of **P1** improved the PLLA elongation at break without compromise to either the Young’s modulus or tensile strength. At 2 wt %, samples showed double the elongation at break and triple the tensile toughness compared to virgin PLLA. The blend also showed nearly equivalent thermal properties to PLLA (*T*_g_ = 58 °C, *T*_c_ = 131 °C, *T*_m_ = 168 °C; Figures S25–S29). These findings are important as they indicate a toughening mechanism different from small-molecule plasticizers which reduce the PLLA *T*_g_. **P1** features alkenes which were reacted with trimethylolpropane tris(3-mercaptopropionate), using azobis(isobutyronitrile) (AIBN) as the initiator.^49,50^ The ratio of [C=C]:[SH] was varied from 1:1, 1:3, and 1:6 to form samples **P2**, **P3**, and **P4**, respectively. The alkene conversion was monitored using ^1^H NMR spectroscopy. **P2** showed 96% conversion, even over prolonged periods or with excess AIBN, whereas **P3** and **P4** both showed quantitative conversion (Figure S30). All the functionalized polymers were purified and dried, and all are amorphous polymers: **P2** shows a *T*_g_ of −8 °C, while **P3** and **P4** show lower values of −15 °C and −32 °C, respectively, attributed to the increasing alkyl chain density (Figures S31–S36).^51^ Raman spectra indicate only **P4** retains thiol resonances, at 2580 cm^–1^, whereas **P2** or **P3** shows complete thiol consumption (Figure S37). The functionalized polyesters were blended with PLLA, at 2 wt % loading, consistent with that used to test **P1**, and samples were subjected to thermal and tensile mechanical evaluation (Table S5 and Figures S21 and S38–S40). **P4** blends showed the best performance with significantly increased elongation at break (25 ± 1.1%) and similar stress at break (43.0 ± 0.4 MPa) to PLLA. **P1** and **P4** were also each blended with a high molar mass commercial PLLA (*M*_n_ = 129 000 g mol^–1^, *Đ* = 1.41, PURASORB PL24 Corbion), which further improved the properties (Table 2 and Figure 4). At 2 wt % of **P4-**PLLA, a PLLA toughness of 17.7 ± 0.21 MJ m^–3^ was observed, which is more than four times higher than PLLA alone. It should be noted that the higher molar mass neat PLLA films (*M*_n_ = 129 000 g mol^–1^) also showed increased tensile strength and elongation at break values compared to the lower molar mass samples (*M*_n_ = 54 600 g mol^–1^). This finding is tentatively attributed to the higher molar mass sample showing greater chain entanglement.^52^

**Table 1:** Tensile Mechanical Data for PLLA Samples with Different Weight Fractions of **P1**^a^

**P1** (wt %)	Young’s modulus (GPa)^b^	tensile strength (MPa)	yield strength (MPa)	elongation at break (%)	tensile toughness (MJ m^–3^)^c^
0 wt %	1.61 ± 0.03	53.1 ± 3.0	57.6 ± 2.6	7 ± 1.2	2.1 ± 0.5
2 wt %	1.61 ± 0.02	45.0 ± 1.5	54.6 ± 2.7	15 ± 0.6	6.4 ± 0.4
5 wt %	1.51 ± 0.03	44.2 ± 1.5	51.4 ± 1.6	13 ± 0.6	5.6 ± 0.4
10 wt %	1.05 ± 0.01	34.4 ± 1.0	35.7 ± 1.7	6 ± 0.2	1.6 ± 0.1
20 wt %	1.12 ± 0.02	23.8 ± 0.5	23.7 ± 0.7	3 ± 0.1	0.39 ± 0.03

a Data measured using blends of commercial PLLA (*M*_n_ = 54 600 g mol^–1^, *Đ* = 1.73) and **P1** (0–20 wt %). Mean values ± std. dev. are calculated from measurements conducted independently on at least three specimens. Polymer tensile specimens were cut from a solvent cast film (2 wt % in CHCl_3_) conforming to dimensions for ISO 527–2 type 5B. Uniaxial tensile measurements conducted at 10 mm min^–1^ extension rate.

b Young’s modulus measured within 0.025–0.25% strain using an external camera.

c Calculated from the area under the stress vs strain plots; errors are the standard deviation of three repeat measurements, using three different specimens cut from the same films.

**Figure 3:**
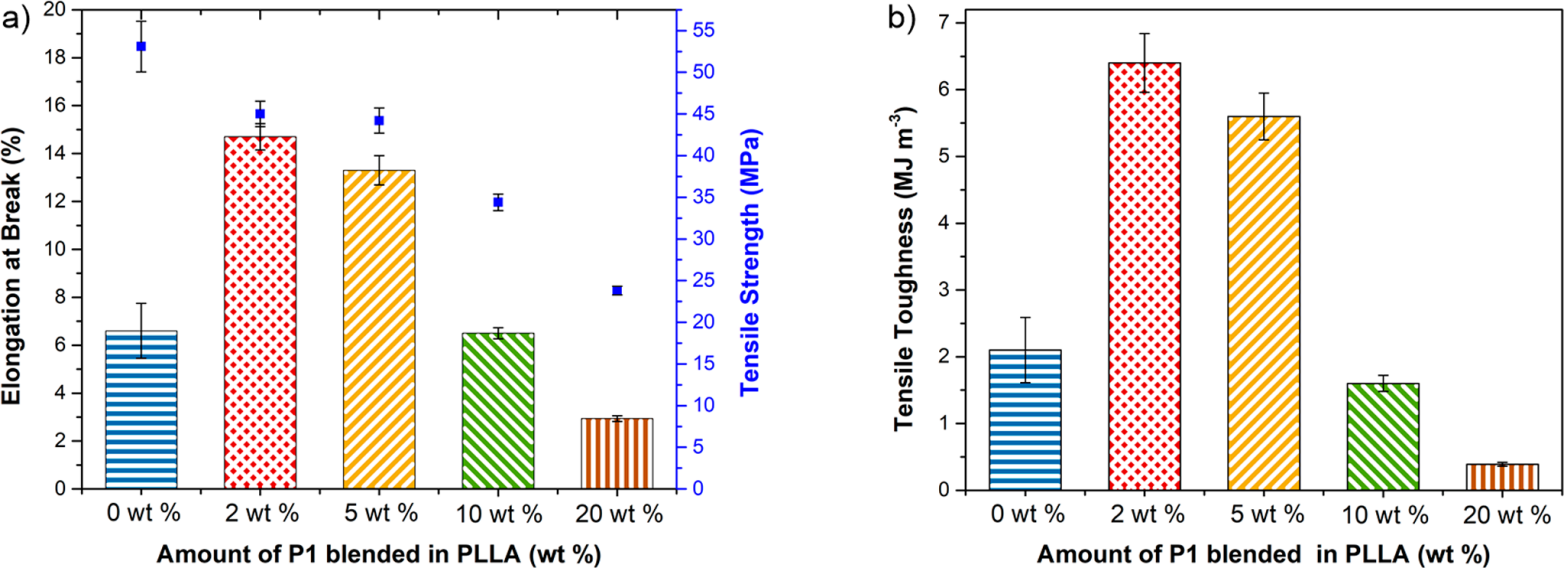
Plots illustrating the different mechanical properties of PLLA (*M*_n_ = 54 600 g mol^–1^, *Đ* = 1.73) samples, toughened with different amounts of **P1**: (a) elongation at break (%) and tensile strength (MPa) (blue squares) vs wt % **P1** and (b) tensile toughness (MJ m^–3^) vs wt % **P1**.

**Table 2:** Tensile Testing Data for High Molar Mass PLLA with 2 wt % Polymer Additives^a^

PLLA additive (2 wt %)	Young’s modulus (GPa)^b^	tensile strength (MPa)	yield strength (MPa)	elongation at break (%)	tensile toughness (MJ m^–3^)^c^
PLLA^d^	2.20 ± 0.40	63.8 ± 0.9	70.9 ± 2.0	9 ± 0.5	4.4 ± 0.2
**P1**	2.20 ± 0.92	56.5 ± 1.5	66.2 ± 3.2	11 ± 0.6	5.7 ± 0.5
**P4**	1.99 ± 0.37	49.6 ± 0.7	58.4 ± 1.0	36 ± 0.9	17.7 ± 0.2

a Data measured using blends of commercial PLLA (*M*_n_ = 129 000 g mol^–1^, *Đ* = 1.41, PURASORB PL24) and **P1** or **P4** (2 wt %). Mean values ± std. dev. are calculated from measurements conducted independently on at least three specimens. Polymer tensile specimens were cut from a solvent cast film (2 wt % in CHCl_3_) conforming to dimensions for ISO 527-2 type 5B. Uniaxial tensile measurements conducted at 10 mm min^–1^ extension rate.

b Young’s modulus measured within 0.025–0.25% strain using an external camera.

c Calculated from the area under the stress vs strain plots; errors are the standard deviation of three repeat measurements, using three different specimens cut from the same films.

d Tensile measurements were conducted using commercial PLLA samples, provided by total Corbion (*M*_n_ = 129 000 g mol^–1^, *Đ* = 1.41, PURASORB PL24) without any additive additions.

**Figure 4:**
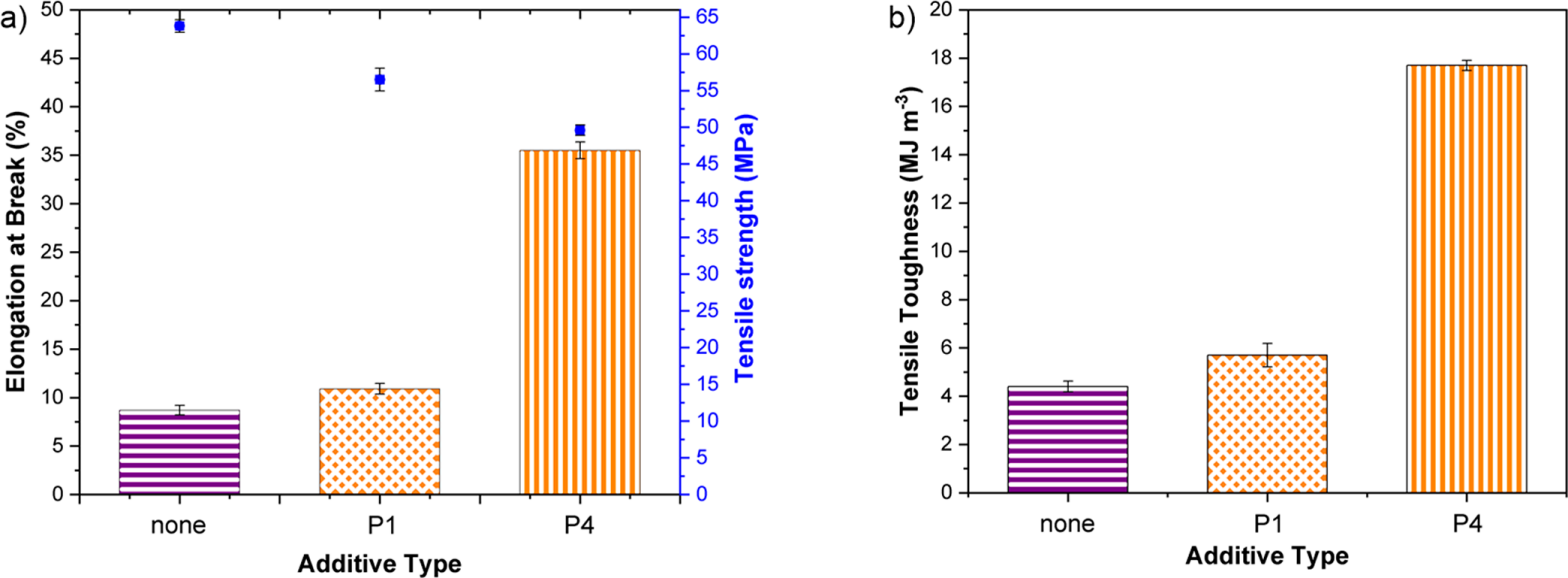
Comparisons of the mechanical properties of high molar mass PLLA (*M*_n_ = 129 000 g mol^–1^, *Đ* = 1.41, PURASORB PL24) with/without 2 wt % of additive: (a) elongation at break (%) and tensile strength (MPa) (blue squares) vs additive type and (b) tensile toughness (MJ m^–3^) vs additive type.

These preliminary mechanical results will require further optimization; nonethless, comparisons against other approaches to toughen or plasticize PLLA reveal their benefits.^53^ For example, small-molecule plasticizers deliver good performances, and among these, tributyl citrate, blended at 10–20 wt %, increases elongation at break to several hundred percent but with the penalty of an 80–90% reduction in Young’s modulus and tensile strength (Table S6).^54−56^ Unfortunately, such blends are also characterized by a reduced PLLA *T*_g_ which increases chain mobility, accelerates cold crystallization, and results in plasticizer exclusion with loss of properties.^55^ An alternative strategy is to blend elastomeric polymers, e.g., PEG, PCL, LDPE, or ABN, with PLLA to produce PLLA matrices containing rubbery particle stress modulator sites.^53,57^ The limitation is the need to tightly control the particle sizes; larger particles cavitate to produce failure sites, and smaller particles fail to improve properties. Additionally, few commercial elastomeric polymers are miscible with PLLA which necessitates adding compatibilizers, complicates formulation, and challenges long-term blend stability.^11^ Recently, Bates and co-workers reported impressive performances using block polyethers, PEG-*b*-PBO, which when optimized at 2 wt % show 20 times greater toughening and 30 times higher elongation at break compared to neat PLLA.^53,57^

It is proposed that these new polyesters, prepared using switchable catalysis, provide rubber toughening since they feature low glass transition temperature PLLA miscible blocks. The polymers show promising performances and, in the future, can be easily modified to deliver further improvements. For example, both polymer structures and processing methods could be fine-tuned to capitalize on the attractive low loadings, delivering toughening.

In conclusion, the efficient route, using an industry relevant catalyst and commercially available monomers, delivers copolymers with tunable properties and excellent PLLA blend performances even at just 2 wt %. Future work will apply the switchable catalysis to other lactones, e.g., ε-caprolactone or β-butyrolactone; anhydrides, e.g., succinic or itaconic; and heterocumulenes, e.g., carbon dioxide, to toughen other commercial polyesters and carbonates. Explorations of ester hydrolyses and, in the future, detailed degradation studies may allow these materials to solve some existing environmental and end-life bioplastics challenges.

## Supporting Information Available

Experimental details, protocols for polymer synthesis, and additional polymerization results including kinetic analyses, polymer characterizations, and polymer thermal and mechanical property studies (PDF)
