# Supplementary material for: Switchable Polymerization Catalysis Using a Tin(II) Catalyst and Commercial Monomers to Toughen Poly(l-lactide)
Source: ACS Macro Lett. 2021 Jun 8;10(7):774–9. doi: 10.1021/acsmacrolett.1c00216 (PMC8296665; doi:10.1021/acsmacrolett.1c00216)
Supplement: Supplementary file 1 — mz1c00216_si_001.pdf [file mz1c00216_si_001.pdf]

Supporting Information

for

Switchable Polymerization Catalysis Using a Tin(II) Catalyst and Commercial Monomers to Toughen Poly(*L*-lactide)

Nattawut Yuntawattana, Georgina L. Gregory, Leticia Peña Carrodeguas, and Charlotte K. Williams*

Chemistry Research Laboratory, Department of Chemistry, University of Oxford, 12 Mansfield Road, Oxford, OX1 3TA, U.K.

* E-mail: charlotte.williams@chem.ox.ac.uk

Table of Contents

S1. General Procedures S4

S2. Instruments S4

S2.1. NMR Spectroscopy S4

S2.2. Gel Permeation Chromatography S4

S2.3. Differential Scanning Calorimetry S4

S2.4. Thermal Gravimetric Analysis S4

S2.5. Tensile Measurement S4

S3. Polymerization Procedures S5

S3.1. Ring-Opening Copolymerization of PO and MA S5

S3.2. Ring-Opening Polymerization of *L*-LA S5

S3.3. One-Pot Polymerization of PO, MA, and *L*-LA S5

S3.4. *In situ* ^{1}H NMR Investigation of the PO/MA/*L*-LA One-Pot Polymerization S5

S3.5. Polymer Purification S5

S3.6. Preparation of Polymer Films for Tensile Testing S5

S4. Additional Polymerization Results for *L*-LA ROP and PO/MA ROCOP S6

S4.1. NMR Spectroscopic data for PE S6

S4.2. Polymerization data Using $Sn(OMe)_2$ S8

S4.3. PO/MA ROCOP data of $Sn(OMe)_2$ and Previously Reported Catalysts S9

S4.4. DSC Data for PE and PLLA S10

S4.5. TGA Data for PE and PLLA S11

S5. Additional Polymerization Results for PO, MA, and *L*-LA Switch Catalysis S12

S5.1. NMR Spectroscopic Data for **P1** S12

S5.2. PO, MA, and *L*-LA Switchable Catalysis data S15

S5.3. T_g Value Determined for **P1** Using the Fox Equation S16

S5.4. DSC Data for **P1** S16

S5.5. TGA Data for **P1** S17

S5.6. Kinetic Analyses for PO/MA/*L*-LA One-Pot Polymerization S18

S5.7. Proposed Catalytic Cycles for Switch Catalysis Using the Sn(II) Catalyst S18

S6. Polymer Film Characterizations and Mechanical Property Studies S20

S6.1. TGA Data for PLLA Films with Different Weight Fractions of **P1** S20

S6.2. Tensile Testing Data S20

S6.3. DSC Data for PLLA Films with Different Weight Fractions of **P1** S25

S6.4. NMR Spectroscopic Data for Functionalized **P2–P4** S28

S6.5. DSC Data for Functionalized Polymers **P2–P4** S29

S6.6. TGA Data for Functionalized Polymers **P2–P4** S30

S6.7. Raman Spectra S32

S6.8. DSC Data for PLLA Films with Two Weight Percent of **P2–P4**. S33

S7. Comparative Data for Toughened PLLA Samples in the Literature S35

S8. References S37

S1. General Procedures

All chemical reagents were purchased from commercial sources (Aldrich, Fisher and Fluorochem) and used as received unless stated otherwise. All solvents used in the reactions were collected from solvent purification system (SPS), degassed three times using freeze-pump thaw process and stored over molecular sieves before use. $Sn(OMe)_2$ was purchased from Santa Cruz biotechnology and used as received. Propylene oxide (PO) was fractionally distilled, over NaH/MeI and under a nitrogen atmosphere, and degassed, three times using a freeze-pump-thaw method, prior to use. Maleic anhydride (MA) was recrystallized from hot chloroform (anhydrous) and sublimed before use. *L*-Lactide (*L*-LA) which was provided free of charge by Total Corbion, was recrystallized from hot anhydrous toluene and sublimed three times prior to use. Benzyl alcohol (BnOH) was fractionally distilled, over CaH_2, and degassed prior to use. All polymerizations were prepared in the nitrogen-filled glovebox (O_2 and water levels < 0.1 ppm) and heated in air-tight vials equipped with a magnetic stir bar.

S2. Instruments

S2.1. NMR Spectroscopy: NMR spectra were recorded on Bruker AVIII HD Nanobay 400 MHz, Bruker AVIII 500 MHz and Bruker AVIII 500 MHz (with ^{13}C cryoprobe) NMR spectrometers.

S2.2. Gel Permeation Chromatography: The molar masses and dispersity values were recorded on an Agilent PL GPC-50 instrument, with HPLC grade $CHCl_3$ as the eluent at a flow rate of 1.0 mL min^{-1} at 30 °C. Two Polymer labs Mixed D columns were used in series. Near monodispersed polystyrene standards were used to calibrate the instrument. The polyesters were dissolved in HPLC grade $CHCl_3$ and filtered prior to analysis.

S2.3. Differential Scanning Calorimetry: DSC data were recorded on DSC3+ (Mettler Toledo, Ltd). A sealed empty crucible was used as a reference, and the DSC was calibrated using indium. Samples were heated from room temperature to 200 °C, at a rate of 10 °C $min^{–1}$, under N_2 flow (100 mL $min^{–1}$). Subsequently, the samples were cooled to –80 °C, at a rate of 10 °C $min^{–1}$ (except samples, containing PLLA where the samples were only cooled down to –20 °C), followed by a heating procedure from –80 °C (or –20 °C for PLA containing samples) to 200 °C, at a rate of 10 °C $min^{–1}$. Each sample was run for two heating–cooling cycles. The glass transition (T_g) and melting (T_m) temperatures reported are taken from the second heating cycle.

S2.4. Thermal Gravimetric Analysis: TGA results were collected from Mettler-Toledo Ltd TGA/DSC 1 system. Powder polymer samples were heated from 30 to 500 °C at a rate of 5 °C $min^{–1}$, under N_2 flow (100 mL $min^{–1}$).

S2.5. Tensile Measurement: Dumbbell specimens were cut according to ISO 527-2, specimen type 5B with Zwick ZCP020 cutting press (length= 35 mm, gauge length = 10 mm, width = 2 mm). Monotonic uniaxial extension experiments were carried out on a Shimadzu EZ-LZ Universal testing instrument using an extension rate of 10 mm $min^{–1}$. An external camera was used to calculate the Young's Modulus, *E* within the 0.025–0.25% strain region. 3 Specimens were tested for each material.

S3. Polymerization Procedures

S3.1. Ring-Opening Copolymerization of PO and MA

The polymerization vessels were charged with maleic anhydride (0.70 g, 7.15 mmol) and $Sn(OMe)_2$ (12.90 mg, 0.071 mmol). Benzyl alcohol (14.75 μL, 0.14 mmol) was then added to a reaction mixture, followed by propylene oxide (5 mL, 71.5 mmol), such that the [MA] = 1.43 M and [Sn] = 14.27 mM. The polymerization vessel was sealed, with PVC tape, removed from the glovebox and heated, at 45 °C, on a preheated aluminium block. Aliquots were withdrawn from the reaction mixture at desired times and quenched with wet $CDCl_3$ (~ 0.6 mL). Monomer conversion and the percentage ester linkages were analysed by ^1H NMR spectroscopy. Polymer molar masses were analysed by GPC.

S3.2. Ring-Opening Polymerization of *L*-LA

A pre-died polymerization vessel, equipped with a magnetic stir bar, was charged with *L*-LA (1.03 g, 7.15 mmol) and $Sn(OMe)_2$ (12.90 mg, 0.071 mmol). Benzyl alcohol (14.75 μL, 0.14 mmol) was then added to the reaction mixture, followed by propylene oxide (5 mL, 71.5 mmol). The polymerization vessel was sealed, with PVC tape, removed from the glovebox and heated, at 45 °C, on a preheated aluminium block. Aliquots were withdrawn from the reaction mixture at desired times and quenched with wet $CDCl_3$ (~ 0.6 mL). Monomer conversion was determined by ^1H NMR spectroscopy. Polymer molar masses were analysed by GPC.

S3.3. One-Pot Polymerization of PO, MA, and *L*-LA

A pre-dried 22 mL glass vial with a PTFE-lined thermoset cap, equipped with a magnetic stir bar, was charged with MA (0.98 g, 9.99 mmol), *L*-LA (1.44 g, 9.99 mmol) and $Sn(OMe)_2$ (18.06 mg, 0.10 mmol). Benzyl alcohol (20.67 μL, 0.20 mmol) and PO (7 mL, 99.91 mmol) were then added to the reaction mixture, followed by the addition of the internal standard mesitylene (0.69 mL, 5.0 mmol). The reaction vessel was sealed with PVC tape, removed from the glovebox and heated, to 45 °C, in a preheated silicone bath. Aliquots were withdrawn from the reaction at desired times and quenched with wet $CDCl_3$ (~ 0.6 mL). The crude product was then analysed by ^1H NMR spectroscopy and the polymer molar masses were determined by GPC.

S3.4. *In situ* ^1H NMR Investigation of the PO/MA/*L*-LA One-Pot Polymerization

A pre-dried J. Young NMR tube was charged with MA (0.07 g, 0.71 mmol), *L*-LA (0.10 g, 0.71 mmol) and $Sn(OMe)_2$ (4.30 mg, 0.02 mmol). Benzyl alcohol (4.92 μL, 0.05 mmol) and the internal standard mesitylene (49.6 μL, 0.36 mmol) were then added using a Hamilton glass syringe. PO (0.50 mL, 7.14 mmol) and $CDCl_3$ (2.29 mL) were added to the reaction mixture such that [MA] = [LA] = 0.25 M and [PO] = 2.5 M). The reaction was then heated at 45 °C and NMR signals were recorded on the Bruker Avance III HD Nanobay 400 MHz NMR instrument. The collected data were analysed comparing the conversions of the different monomers against the internal standard.

S3.5. Polymer Purification

The crude reaction mixture was dissolved in CH_2Cl_2 and dropwise added into a hexane solution to precipitate the polymer. The polymer was filtered and dried under vacuum. The polymer was re-dissolved in CH_2Cl_2 and rapidly filtered through a small pad of silica. The polymer was then precipitated, following the above procedure, into hexane three times and dried (vacuum oven, 60 °C, 48 h).

S3.6. Preparation of Polymer Films for Tensile Testing

The blends of the test polymers and PLLA were dissolved in $CHCl_3$ (2 wt %) and allowed to stir at room temperature for 4 h or until all polymer was fully dissolved. The polymer solution was then transferred into a PTFE mould. The PTFE mould was covered by aluminium foil and left at room temperature for 20 h to allow the solvent to evaporate. The polymer film was then removed from the mould and dried in a vacuum oven, at 60 °C, for 72–96 h. The remaining solvent residue in the blend materials was checked using ^1H NMR spectroscopy and thermogravimetric analysis before the tensile measurements were conducted.

S4. Additional Polymerization Results for *L*-LA ROP and PO/MA ROCOP

S4.1. NMR Spectroscopic data for PE

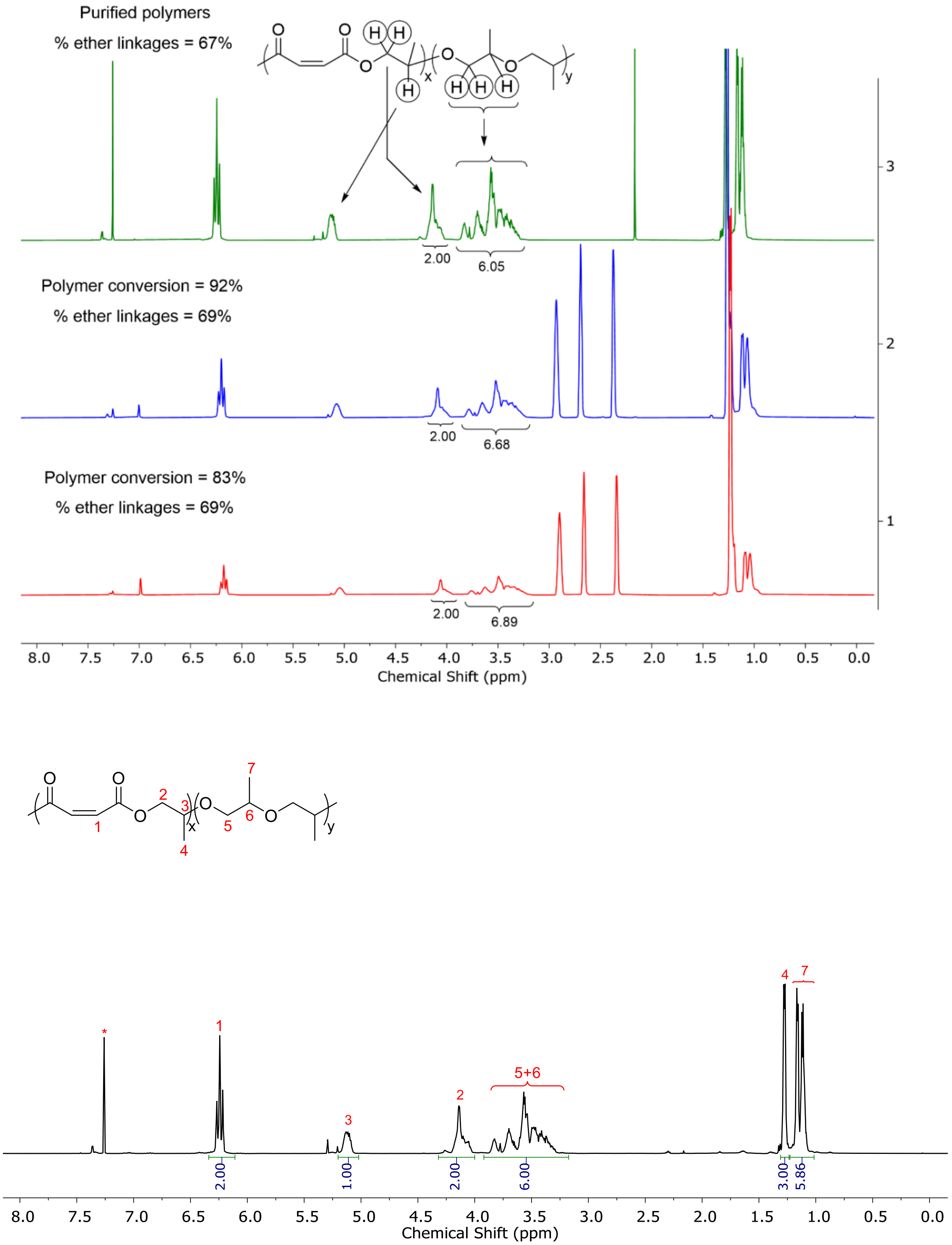

Figure S1. TOP: Stacked ^{1}H NMR spectra showing crude and purified poly(ester-*ran*-ether), PE, at different conversions and BOTTOM: The ^{1}H NMR spectrum of purified PE in $CDCl_3$.

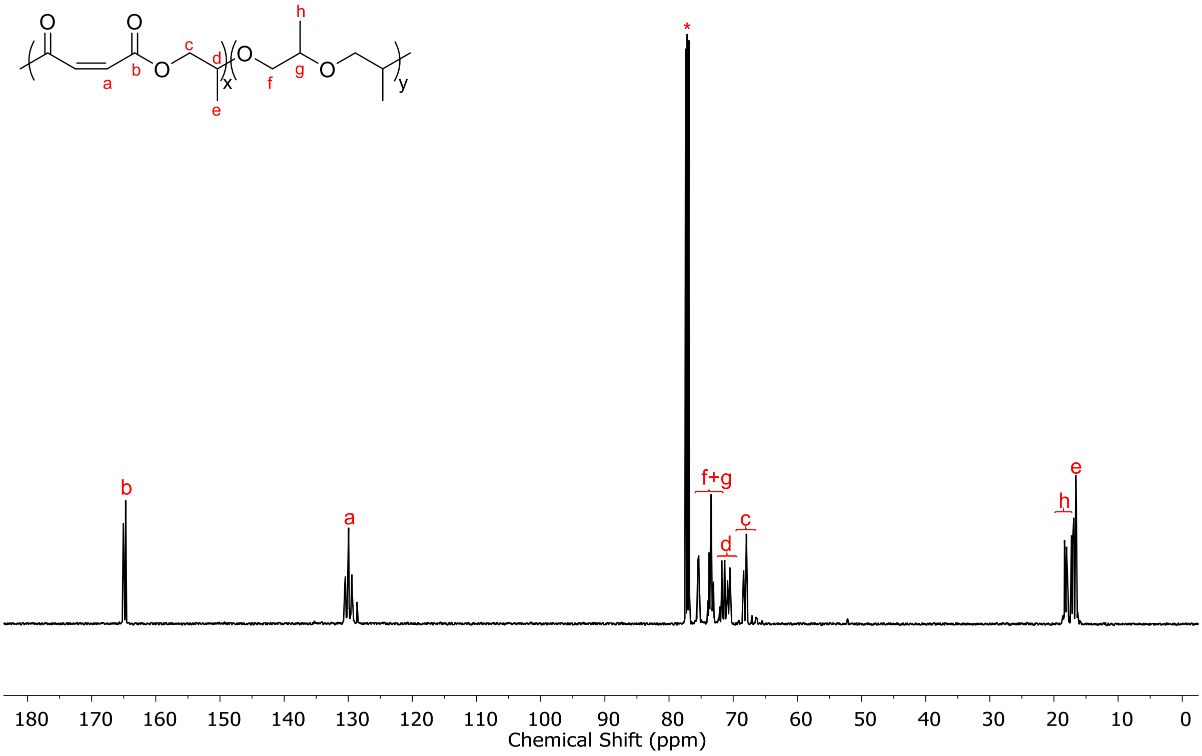

Figure S2. $^{13}C\{^1H\}$ NMR spectrum of poly(ester-*ran*-ether) in $CDCl_3$.

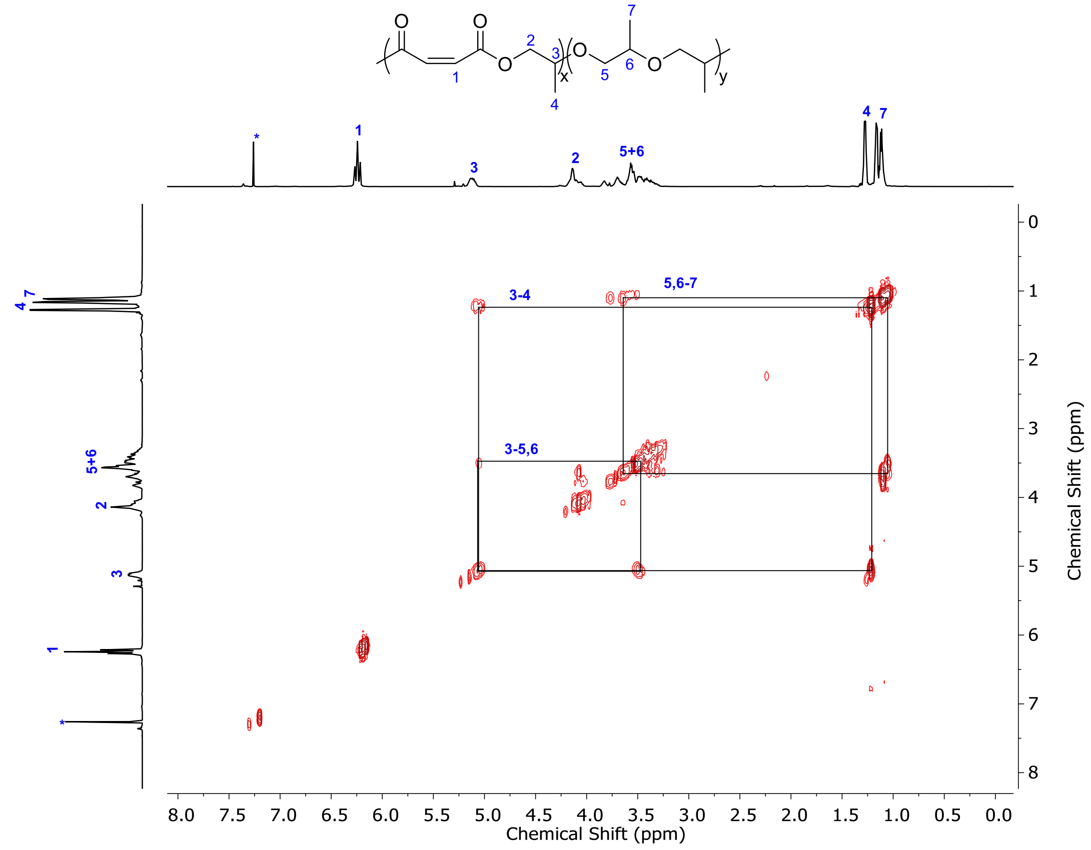

Figure S3. ^1H-COSY NMR spectrum of poly(ester-*ran*-ether) in $CDCl_3$.

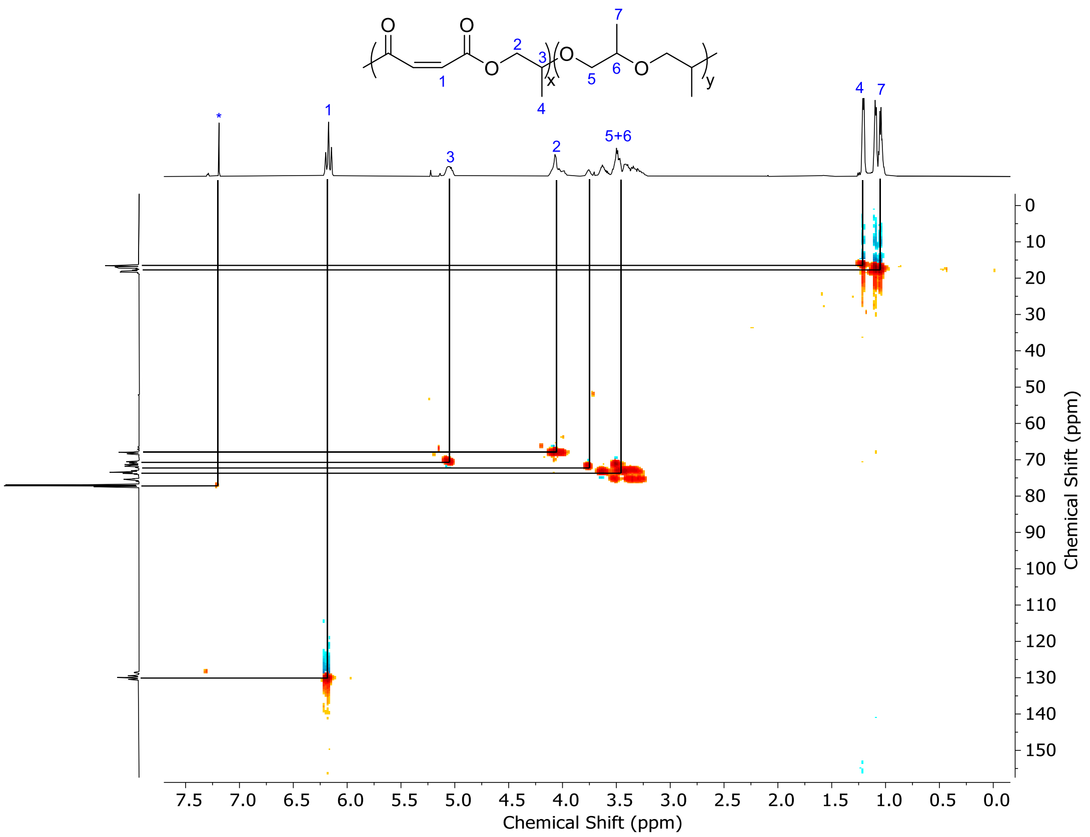

Figure S4. HSQC NMR spectrum of poly(ester-*ran*-ether) in $CDCl_3$.

S4.2. Polymerization data Using $Sn(OMe)_2$

Table S1. Polymerization data for L-LA ROP and PO/MA ROCOP initiated by $Sn(OMe)_2$.[a]

Monomer	Time (h)	Conv. (%)[b]	Ether linkages (%)[c]	$M_{n,GPC}$ (g mol^{-1})[d]	$M_{n,\,calc.}$ (g mol^{-1})[e]	$Đ$[d]	T_g (°C)[f]	T_m (°C)[f]
MA	5	95	67	5 600	5 100	1.25	–19	-
L-LA	3	93	-	4 100	3 400	1.36	-	159

[a] Conditions: polymerizations were conducted at 45 °C in neat PO and [Sn]:[BnOH]:[monomer]:[PO] = 1:2:100:1000 where [monomer] = 1.43 M, [Sn] = 14.3 mM. [b] Determined by comparing the integrals of selected resonances for the anhydride or lactone in the ^1H NMR spectra ($CDCl_3$): MA (7.57-7.48 ppm), poly(ester-*ran*-ether) (6.88-6.63 ppm), L-LA (5.06-4.98 ppm), PLLA (5.22-5.08 ppm). [c] Calculated by comparing the integrals of selected resonances for propylene oxide in the ^1H NMR spectra ($CDCl_3$): ester linkages (5.20-5.03 ppm, methine proton and 4.31-3.95 ppm, methylene proton), ether linkages (3.93-3.17 ppm). [d] Determined by GPC analysis, against polystyrene standards, in $CHCl_3$; for PLLA a correction factor of 0.58 was applied as per the literature.[1] [e] For poly(ester-*ran*-ether), calculated from [((2×58.08) + 98.06) × % Conv.]/4 and for PLLA, calculated from (144.14 × % Conv.)/4. [f] Determined by DSC using a heating rate of 10 °C min^{-1} and where the T_g values are reported from the second heating cycle.

S4.3. PO/MA ROCOP data of $Sn(OMe)_2$ and Previously Reported Catalysts

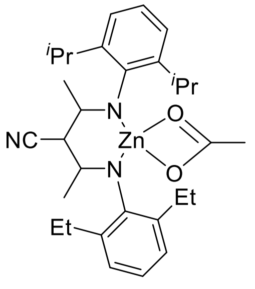

1

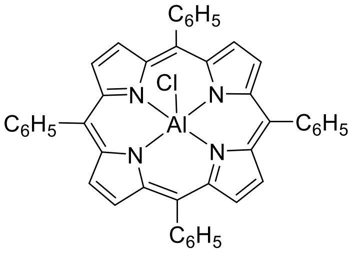

2

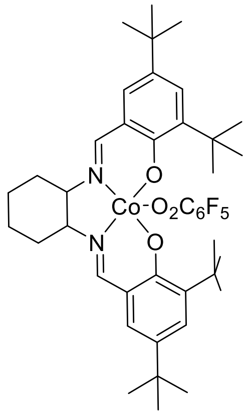

3

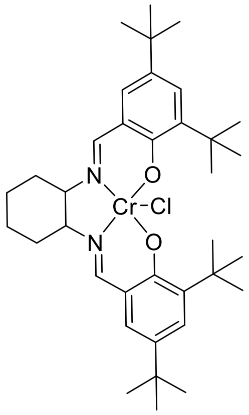

4

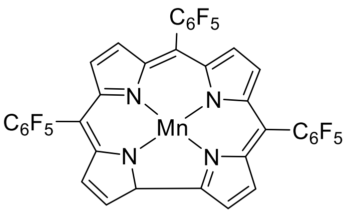

5

Figure S5. Structures of previously reported catalysts for PO/MA ROCOP.[2-4]

Table S2. Summary of PO/MA ROCOP data using $Sn(OMe)_2$ and other previously reported catalysts.

Catalyst	[cat.]:[MA]:[PO]	T (°C)	t (h)	Conv. (%)	Ether linkages (%)	$M_{n,GPC}$ (g mol^{-1})	$Đ$	Ref
$Sn(OMe)_2$[a]	1:100:1000	45	5	95	67	5 600	1.25	This work
$Mg(OEt)_2$[b]	1:122:122	80	48	42	51	4 200	1.17	4
**1**[c]	1:200:200	45	15	5	86	5 000	1.20	3
**2**[c]	1:200:200	45	15	7	50	14 000	1.10	3
**3**[c]	1:200:200	45	15	12	< 1	5 000	1.10	3
**4**[c]	1:200:200	45	15	47	< 1	6 000	1.30	3
**5**[d]	1:100:2000	30	144	50	< 1	3 700	1.20	2

[a] Polymerizations were conducted under solvent free conditions. [b] Polymerizations were conducted in toluene and with [MA] = [PO] = 7.14 M. [c] Polymerizations in toluene and with [MA] = [PO] = 4 mM. [d] Polymerizations were conducted with $PPNOBzF_5$ as the co-catalyst, with [**5**]:[$PPNOBzF_5$] = 1:2 and neat.

S4.4. DSC Data for PE and PLLA

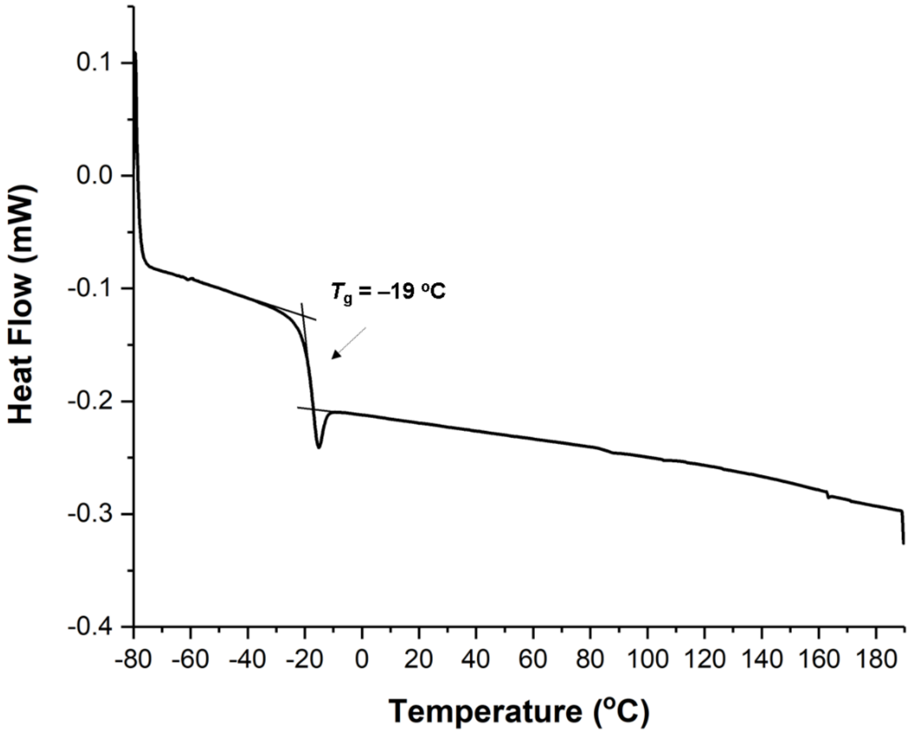

Figure S6. DSC thermogram of poly(ester-*ran*-ether).

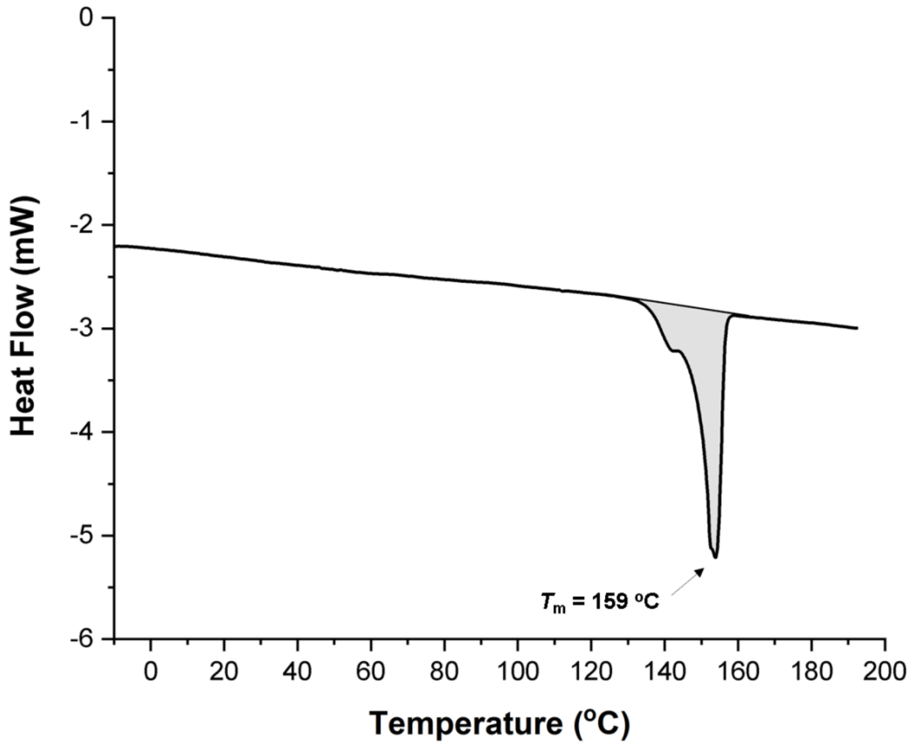

Figure S7. DSC thermogram of PLLA.

S4.5. TGA Data for PE and PLLA

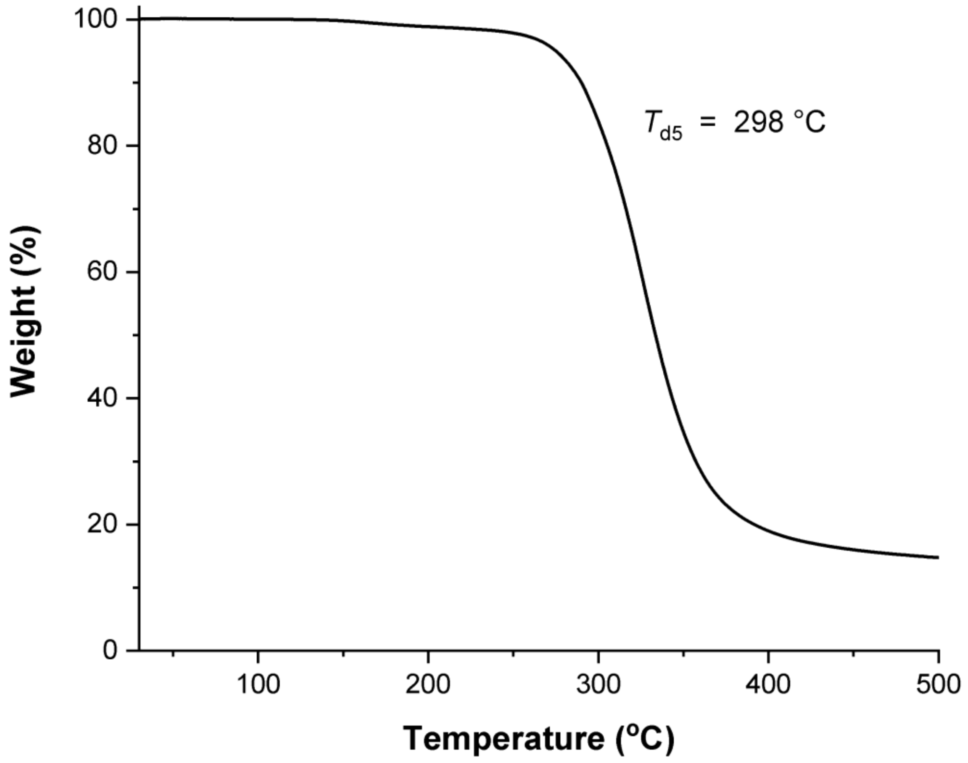

Figure S8. TGA thermogram of poly(ester-r*an*-ether).

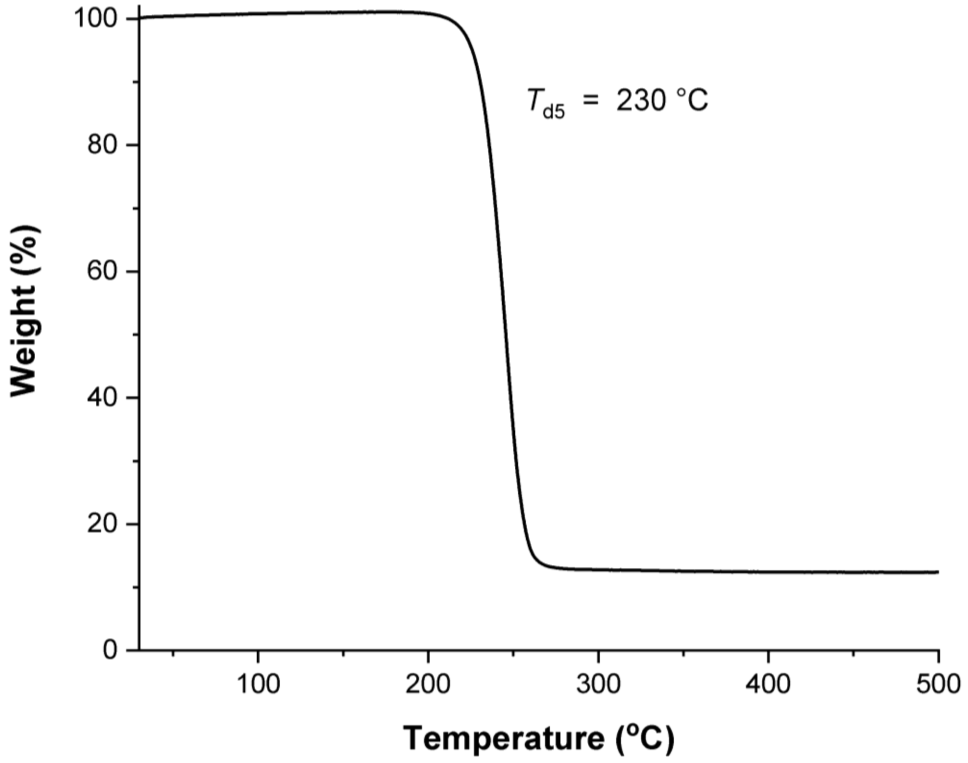

Figure S9. TGA thermogram of PLLA.

S5. Additional Polymerization Results for PO, MA, and L-LA Switch Catalysis

S5.1. NMR Spectroscopic Data for P1

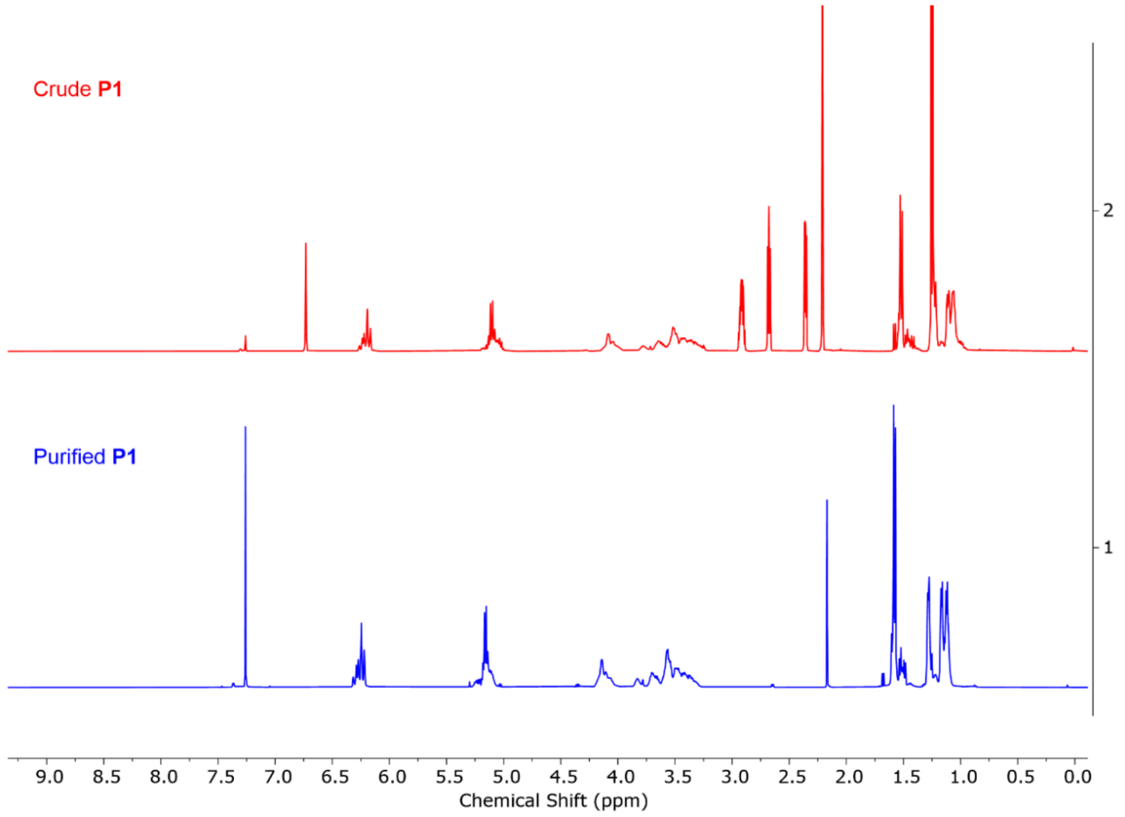

Figure S10. Stacked ^{1}H NMR spectra of purified **P1** (bottom) and crude **P1** (top) in $CDCl_3$.

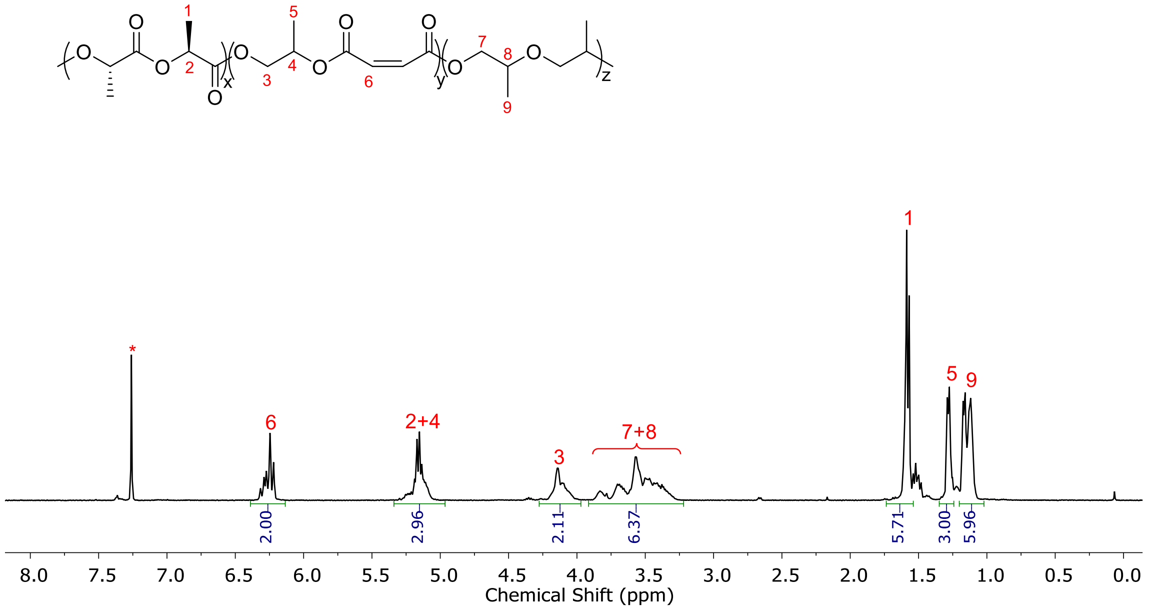

Figure S11. ^{1}H NMR spectrum of **P1** in $CDCl_3$.

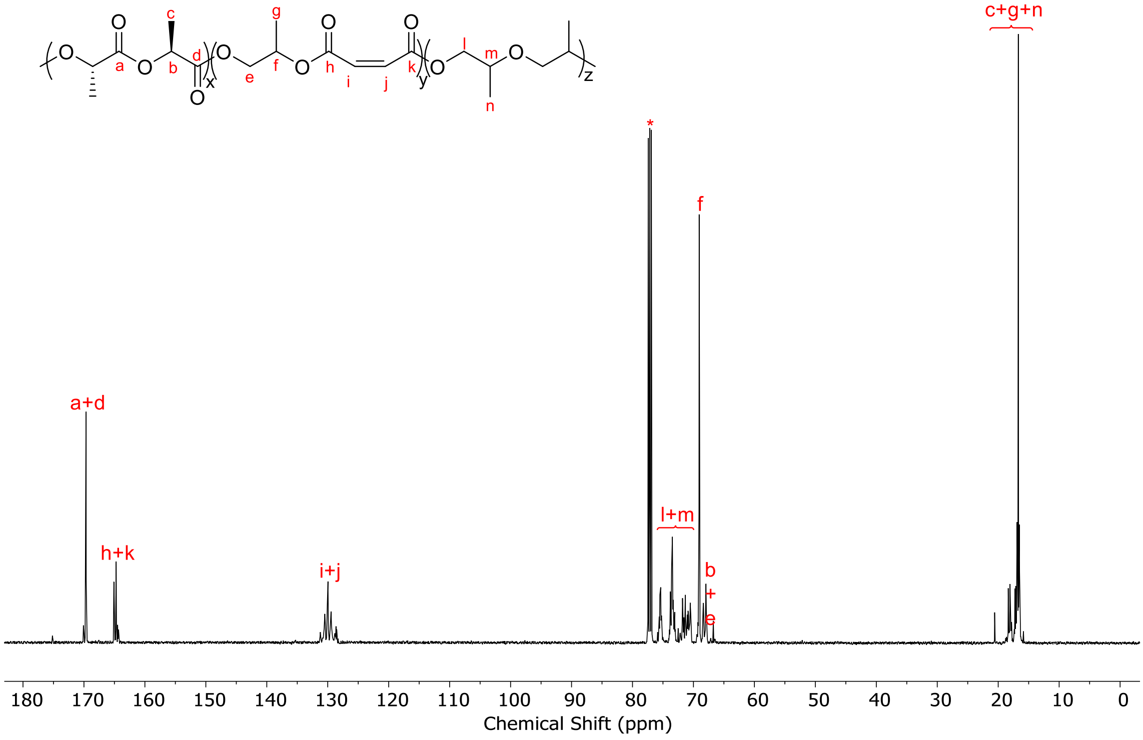

Figure S12. $^{13}C\{^1H\}$ NMR spectrum of **P1** in $CDCl_3$.

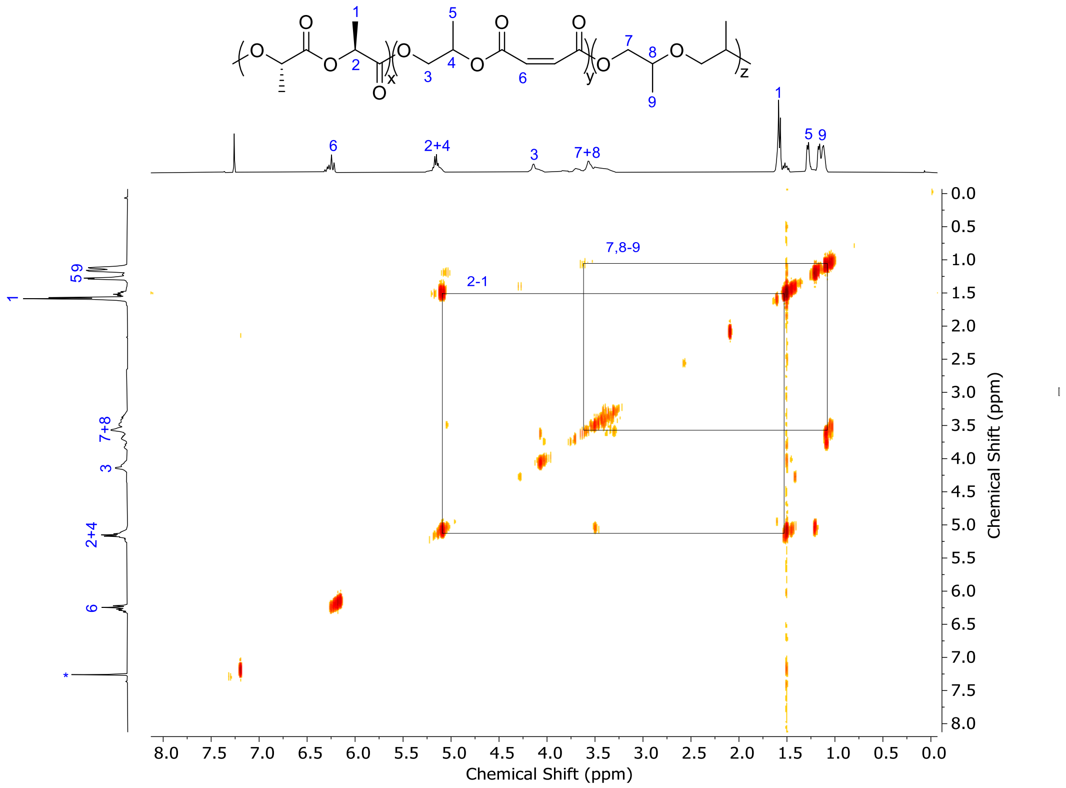

Figure S13. ^{1}H-COSY NMR spectrum of **P1** in $CDCl_3$.

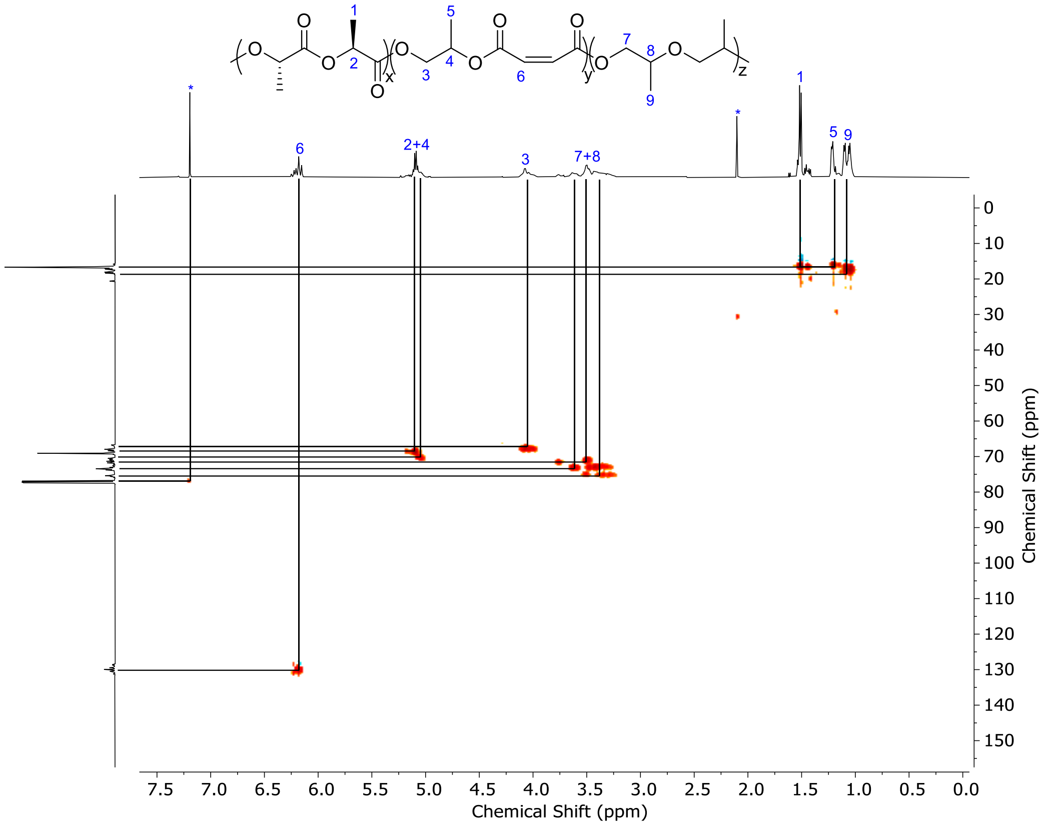

Figure S14. HSQC NMR spectrum of **P1** in $CDCl_3$.

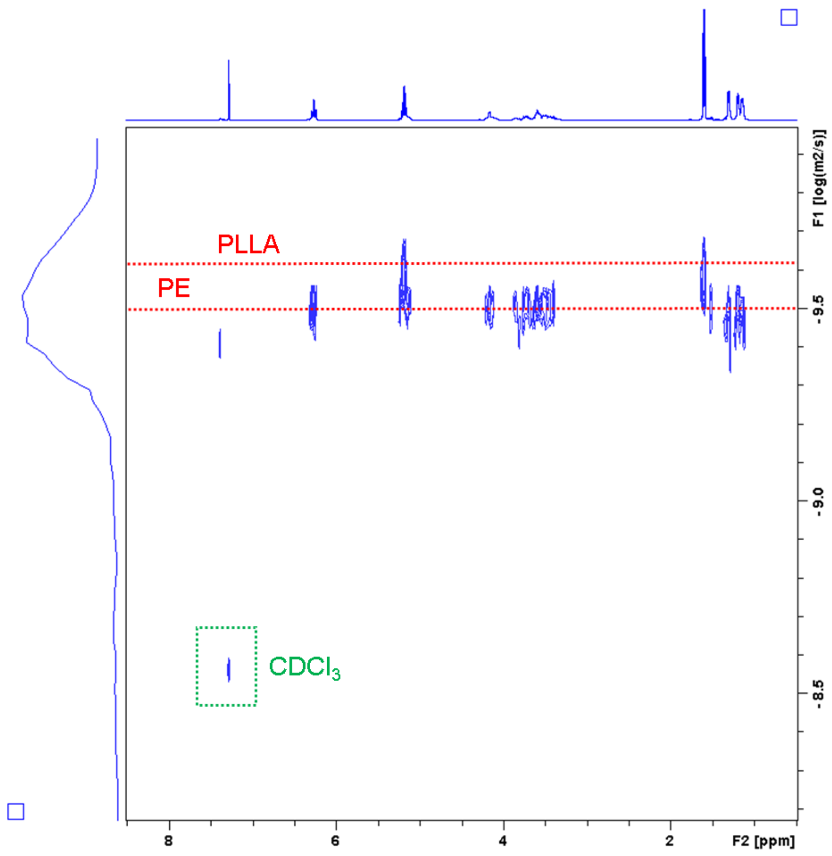

Figure S15. ^{1}H DOSY NMR spectrum of PE and PLLA blend.

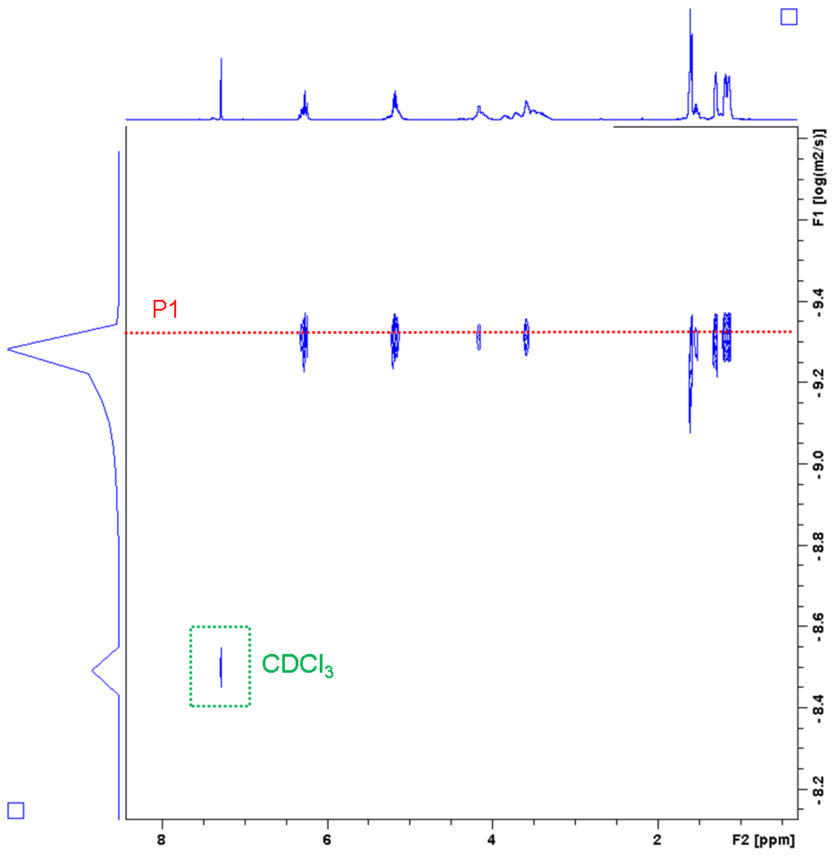

Figure S16. ^{1}H DOSY NMR spectrum of **P1**.

S5.2. PO, MA, and L-LA Switchable Catalysis data

Table S3. Polymerization data from one pot-polymerization of L-LA, PO, and MA using $Sn(OMe)_2$.[a]

$[Sn(OMe)_2]$:[BnOH]: [MA]:[L-LA]:[PO]	**Time (h)**	**% Conv. (MA)**[b]	**% Conv. (LA)**[c]	$M_{n,GPC}$ **(g mol^{-1})**[d]	$Đ$[d]	T_g **(°C)**[e]	T_m **(°C)**[e]
1:2:100:100:1000	105	> 99	93	12 000	1.25	–6	-

[a] Conditions: Polymerizations were conducted at 45 °C, in neat PO, with [L-LA] = [MA] = 1.43 M and $[Sn(OMe)_2]$:[BnOH]:[MA]:[L-LA]:[PO] = 1:2:100:100:1000. [b] Calculated by integration of the methine region of the ^{1}H NMR spectrum (MA, 7.57-7.48 ppm; polymer, 6.88-6.63 ppm). [c] Determined by comparison of the methyl proton resonances for L-LA against the mesitylene internal standard (L-LA, 2.16-2.05 ppm; mesitylene, 2.79-2.70 ppm). [d] Determined by GPC analysis, against polystyrene standards, in $CHCl_3$. [e] Determined by DSC using a heating rate of 10 °C min^{-1} and where the T_g and T_m values are reported from the second heating cycle.

S5.3. T_g Value Determined for P1 Using the Fox Equation

$$\frac{1}{T_g} = \frac{w_1}{T_{g1}} + \frac{w_2}{T_{g2}}$$

$$\frac{1}{T_{gP1}} = \frac{w_{PLLA}}{T_{gPLLA}} + \frac{w_{PE}}{T_{gPE}}$$

Note: The T_g value for PLLA = 58 °C is taken from the literature.[5]

$$\frac{1}{T_{gP1}} = \frac{0.385}{58+273} + \frac{0.615}{(-19)+273}$$

$$\frac{1}{T_{gP1}} = 0.0012 + 0.0024 = 0.0036$$

$$T_{gP1} = \frac{1}{0.0036} = 278\ K = 5\ °C$$

S5.4. DSC Data for P1

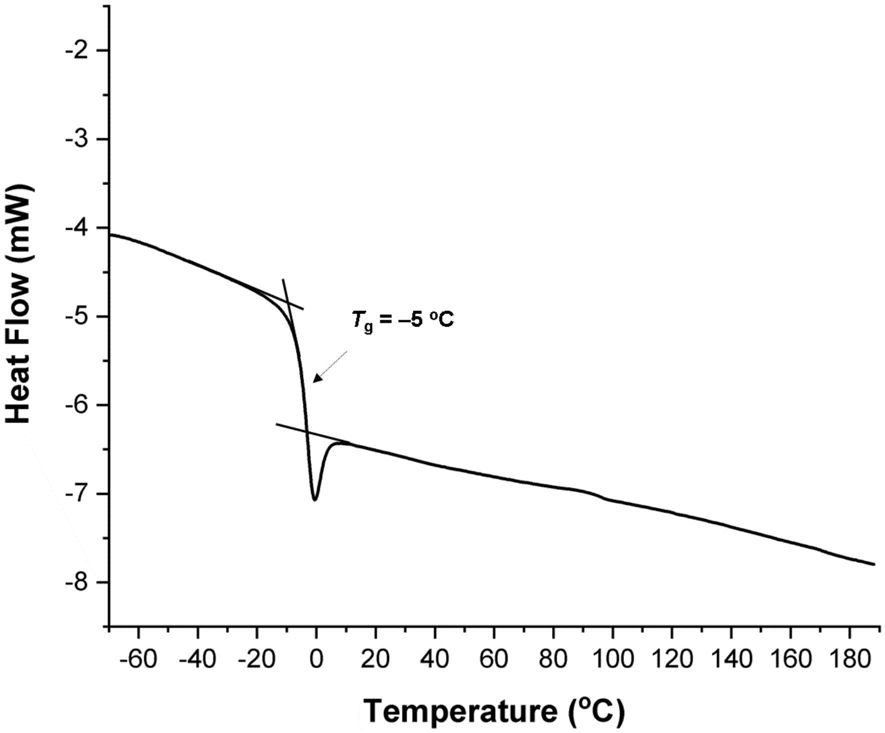

Figure S17. DSC thermogram of **P1**.

S5.5. TGA Data for P1

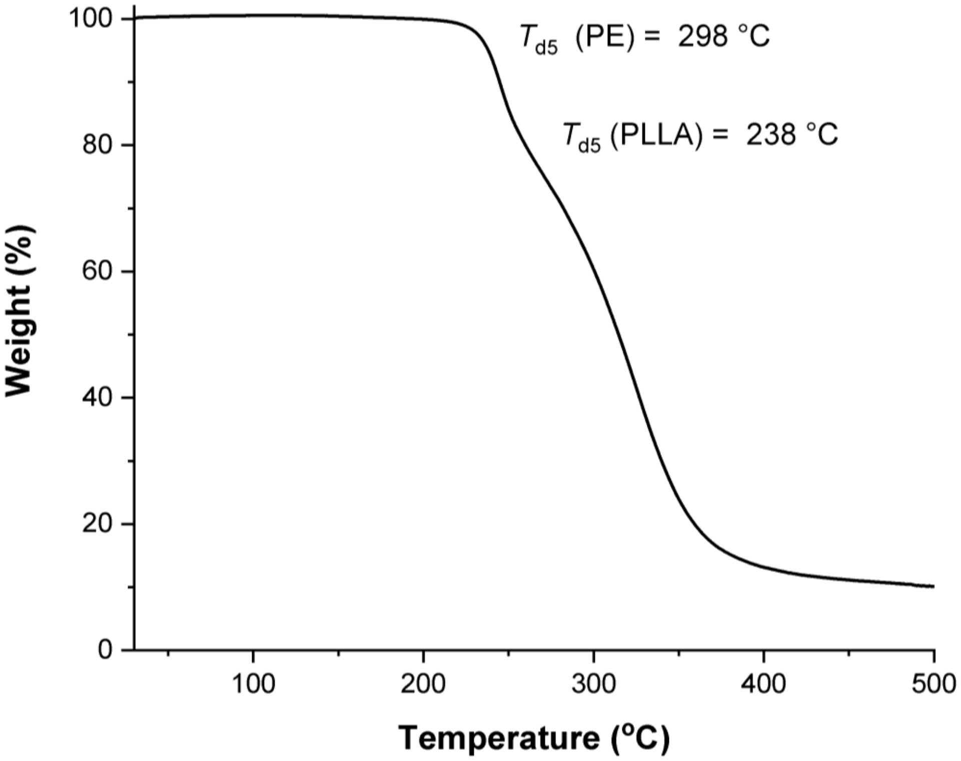

Figure S18. TGA thermogram of **P1**.

S5.6. Kinetic Analyses for PO/MA/*L*-LA One-Pot Polymerization

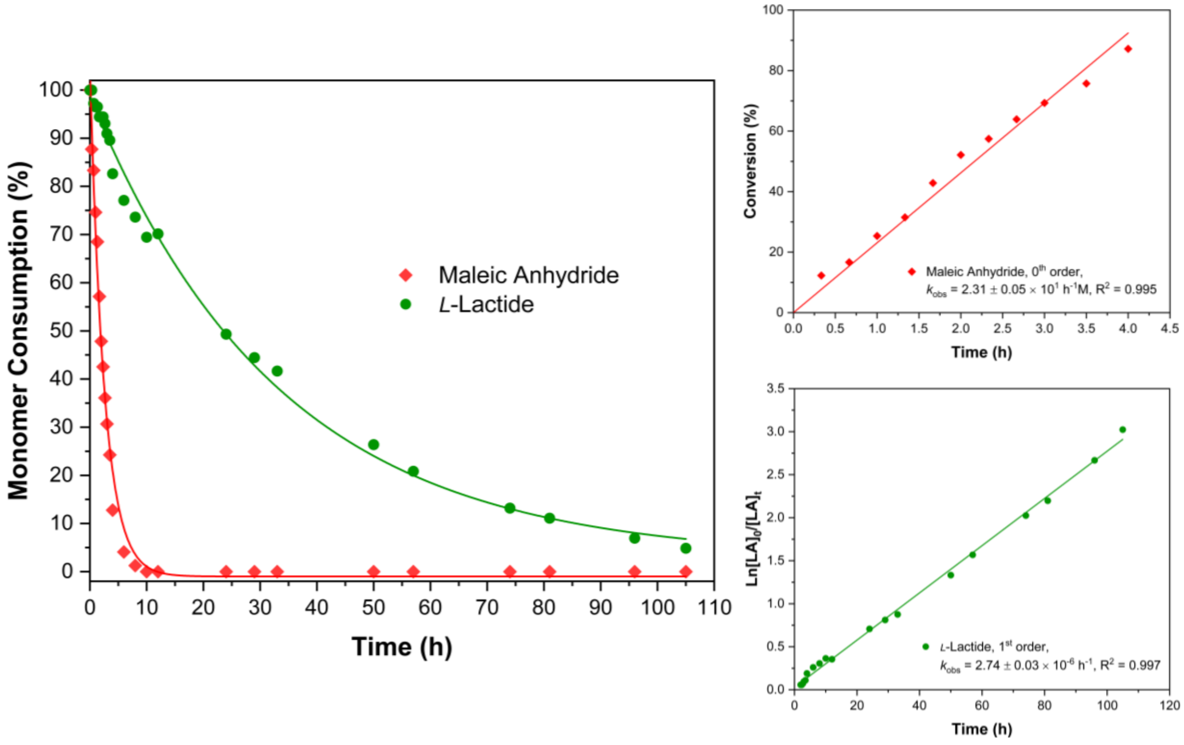

Figure S19. Kinetic measurements from conversion vs time data. Data show MA (red) and *L*-LA (green) conversions vs time, with the associated fits for zeroth order (MA) and first order (*L*-LA) dependences.

S5.7. Proposed Catalytic Cycles for Switch Catalysis Using the Sn(II) Catalyst

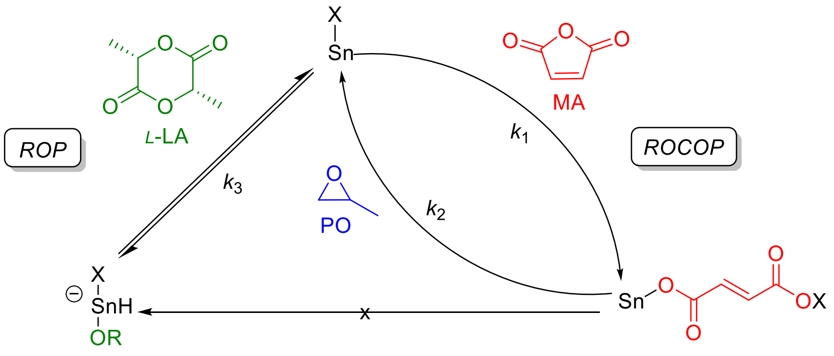

Scheme S1. Switch catalysis reactions using the Sn(II) catalyst.

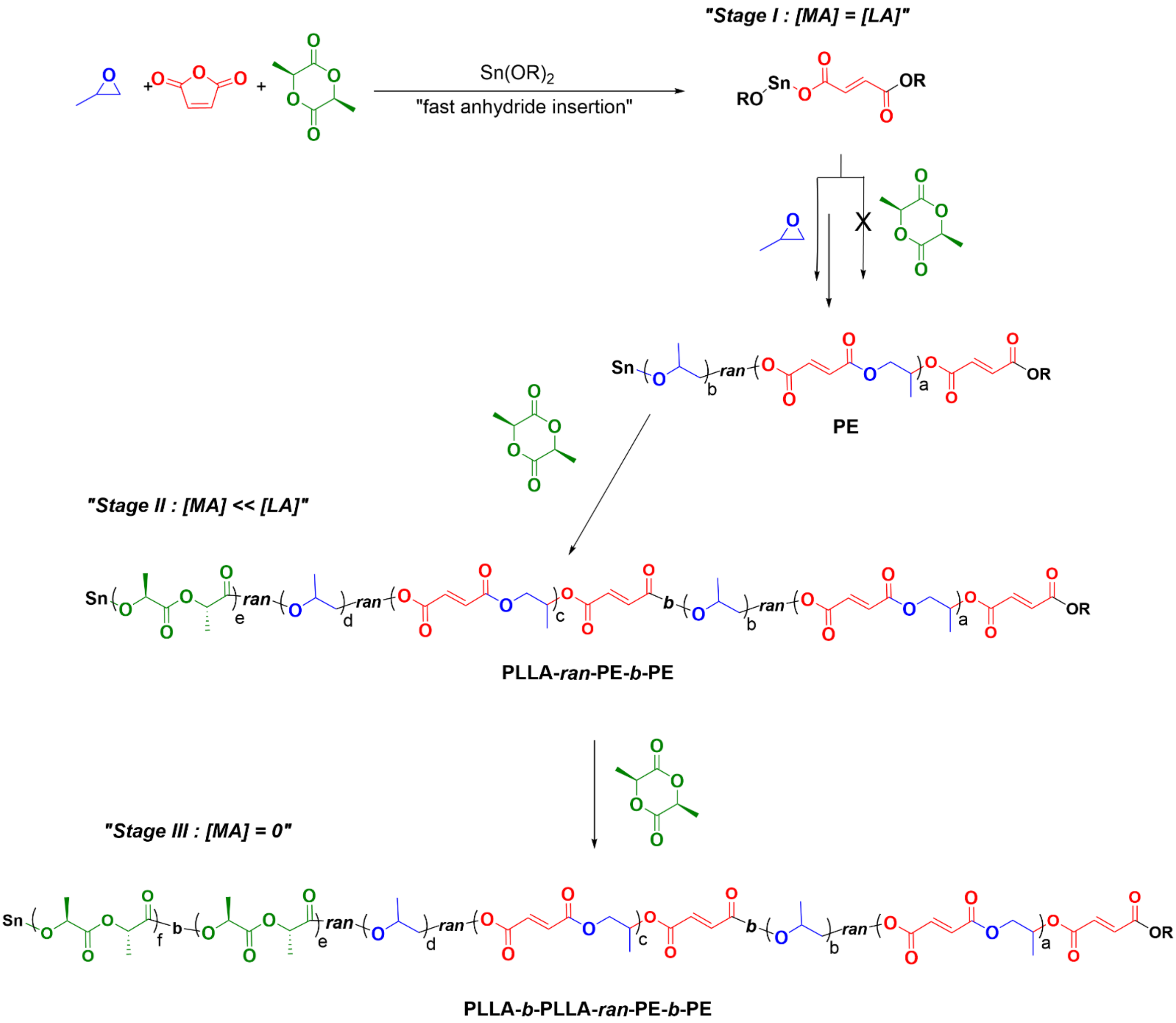

Scheme S2. Synthesis and chemical structure of PLLA-*b*-PLLA-*ran*-PE-*b*-PE *via* three stage 'switch' catalysis.

S6. Polymer Film Characterizations and Mechanical Property Studies

S6.1. TGA Data for PLLA Films with Different Weight Fractions of P1

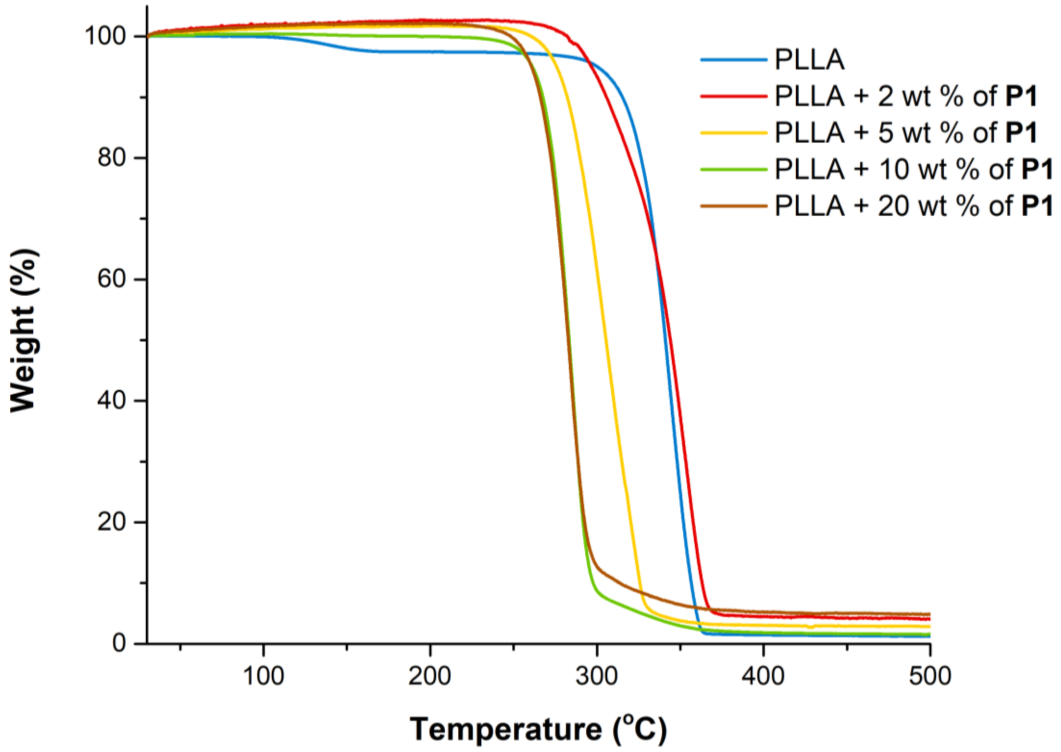

Figure S20. TGA thermogram of blended PLLA film with 0–20 wt % of **P1**.

S6.2. Tensile Testing Data

Table S4. Thermal properties of the polymers.

Polymer	$M_{n,GPC}$ (g mol^{-1})^{d}	$Đ^{d}$	T_g (°C)^{f}	T_m (°C)^{a}	T_{d5} (°C)^{f}
PE	5 600	1.25	–19	-	298
PLLA	4 100	1.36	-	159	230
**P1**	12 000	1.25	–5	-	238, 298
**P2**	11 400	1.39	–8	-	276
**P3**	9 400	1.49	–15	-	292
**P4**	8 300	1.53	–32	-	309

Table S5. Tensile mechanical data for PLLA, with 2 wt % of **P1**, **P2**, **P3**, and **P4**.[a]

Additive	Young's Modulus (GPa)[b]	Tensile Strength (MPa)	Yield Strength (MPa)	Elongation at Break (%)	Tensile Toughness (MJ m^{-3})[c]
**P2**	2.27 ± 0.17	54.3 ± 1.0	60.1 ± 0.4	9 ± 0.5	4.1 ± 0.27
**P3**	1.62 ± 0.02	53.4 ± 2.5	62.8 ± 0.7	15 ± 1.8	7.7 ± 0.95
**P4**	1.72 ± 0.13	43.0 ± 0.4	54.4 ± 1.0	25 ± 1.1	11.1 ± 0.49

[a] Data measured using blends of commercial PLLA (M_n = 54 600 g mol^{-1}, $Đ$ = 1.73) and polymers **P2–P4**. Mean values ± std. dev. are calculated from measurements conducted independently on at least 3 specimens. Polymer tensile specimens were cut from a solvent cast film (2 wt % in $CHCl_3$) conforming to dimensions for ISO 527-2 type 5B. Uniaxial tensile measurements conducted at 10 mm min^{-1} extension rate. [b] Young's modulus measured within 0.025–0.25% strain using an external camera. [c] Calculated from the area under the stress vs strain plots; errors are the standard deviation of three repeat measurements, using three different specimens cut from the same films.

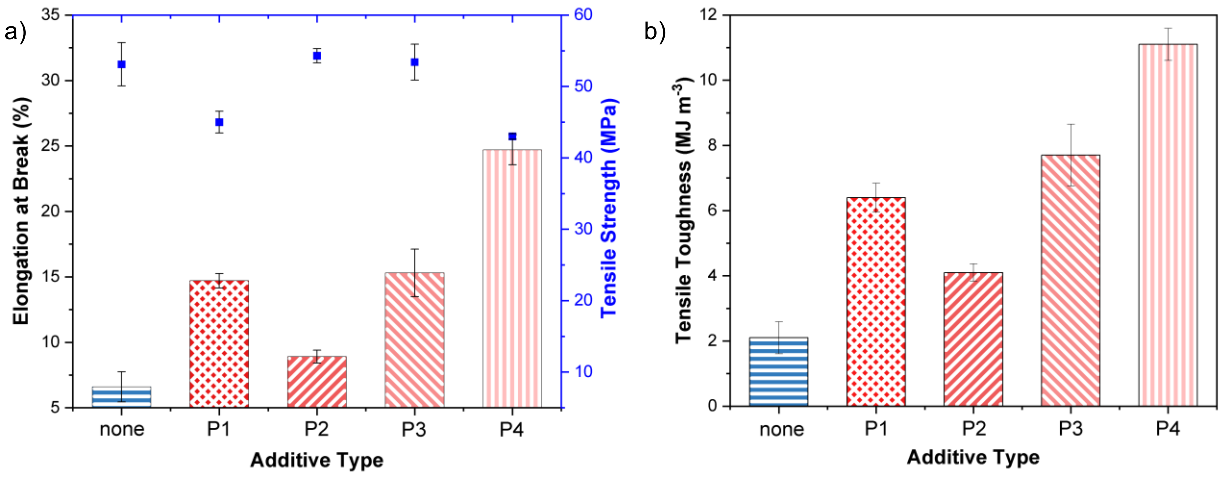

Figure S21. Comparisons of the mechanical properties of PLLA (M_n = 54 600 g mol^{-1}, $Đ$ = 1.73) with/without 2 wt % additive: a) elongation at break (%) and tensile strength (MPa) (blue squares) vs additive type and b) tensile toughness (MJ m^{-3}) vs additive type.

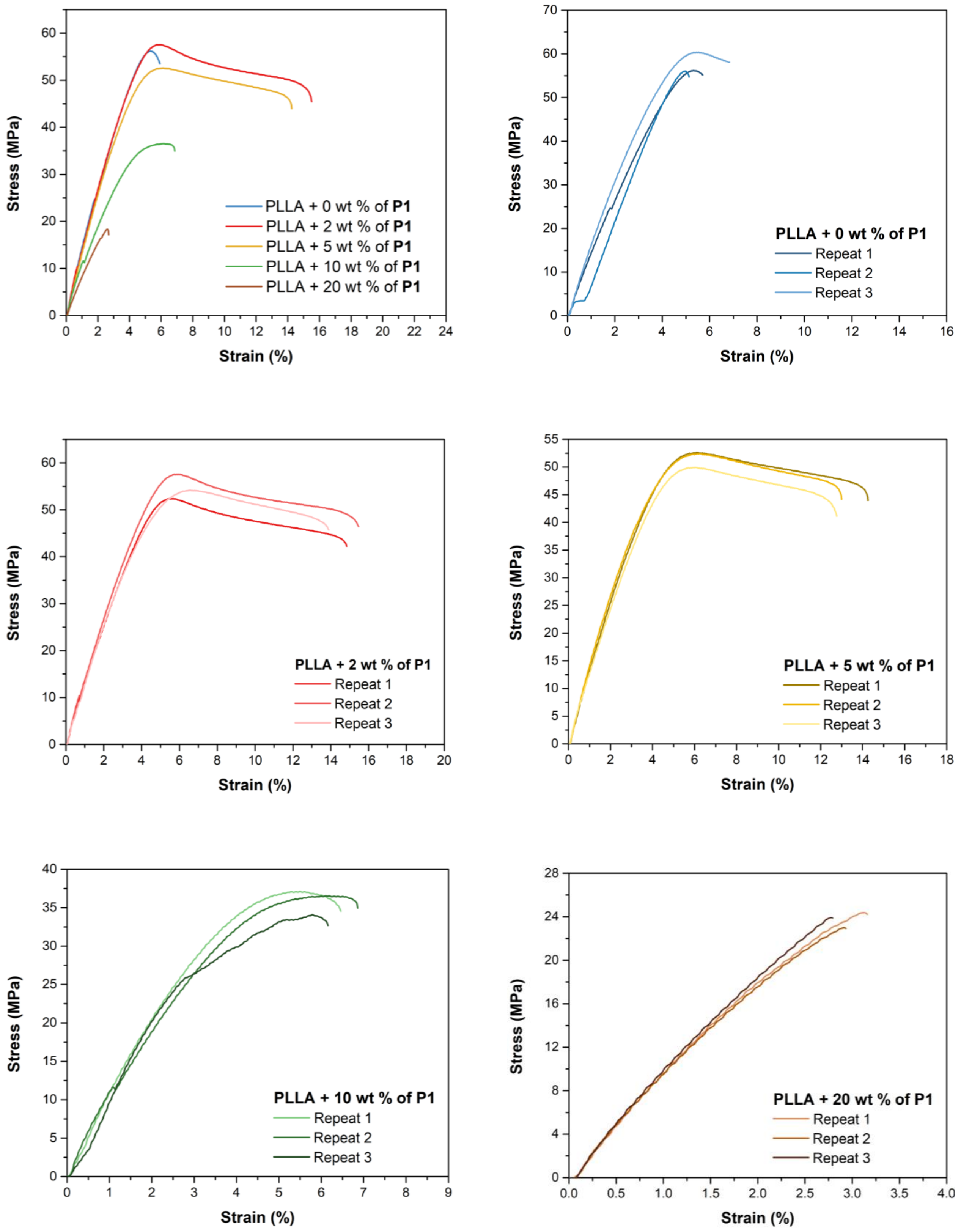

Figure S22. Plots of Stress *vs.* Strain for PLLA, blended with 2–20 wt % **P1**.

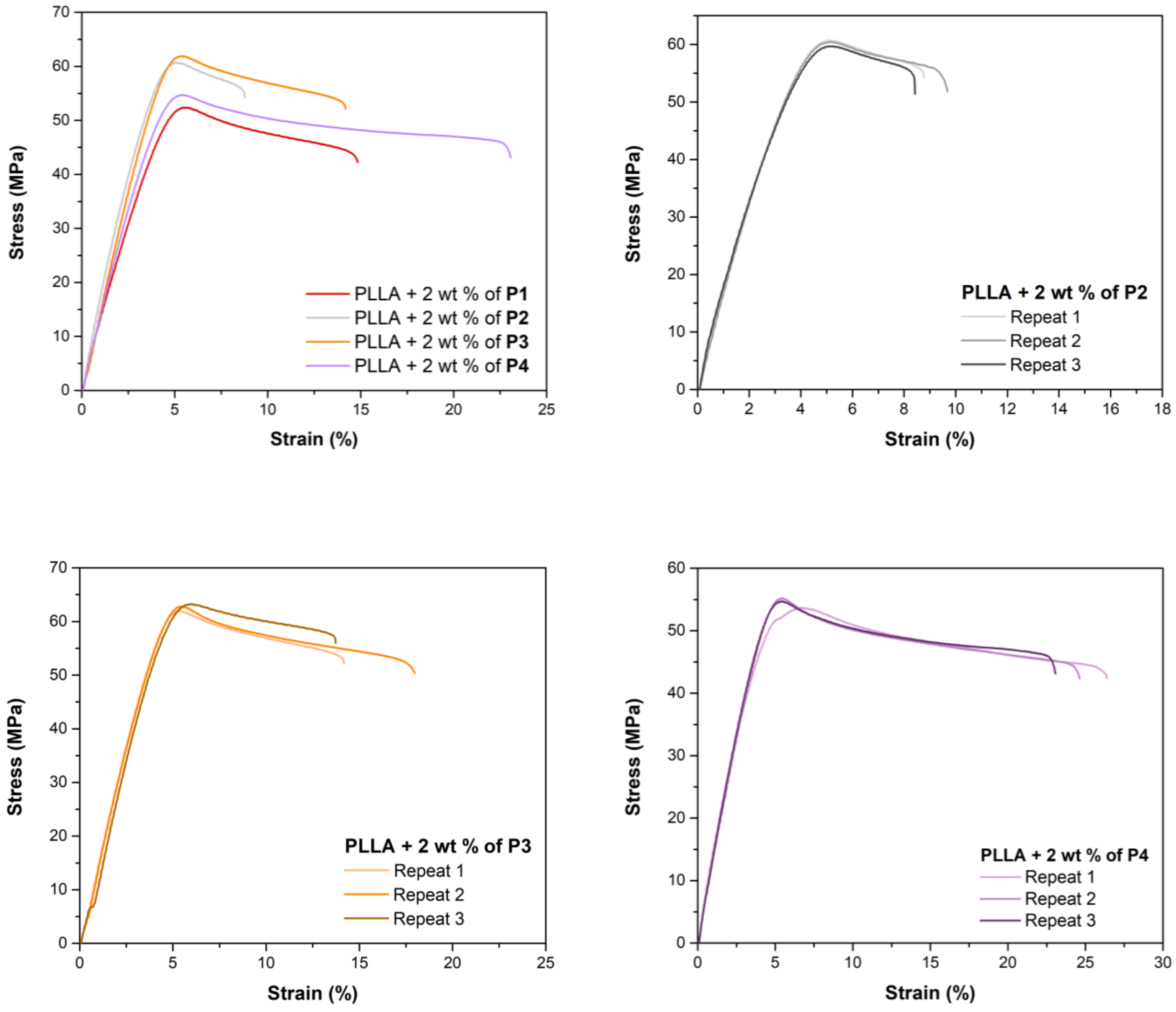

Figure S23. Plots of Stress against Strain for PLLA, blended with 2 wt % of **P1**, **P2**, **P3**, and **P4**.

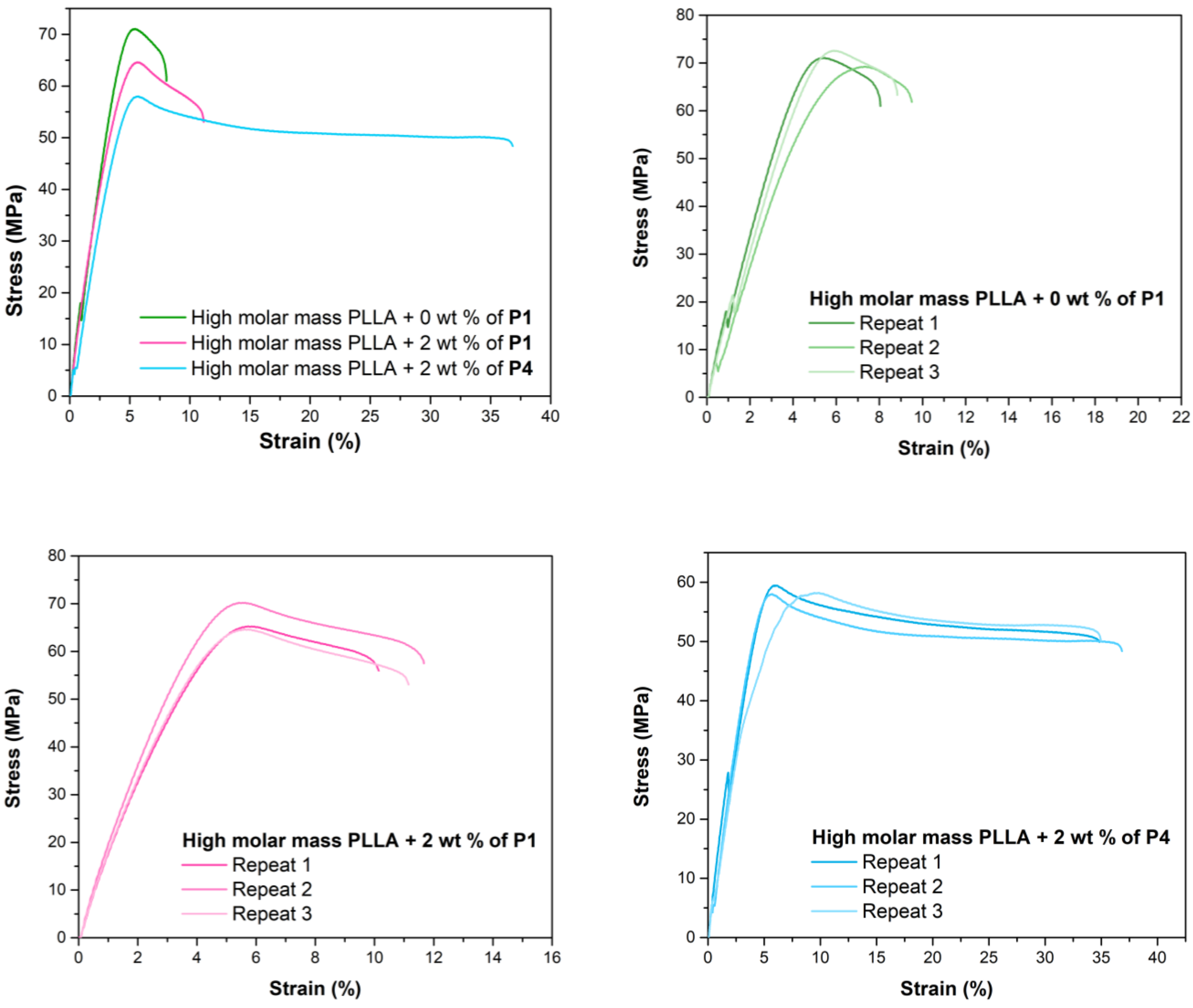

Figure S24. Plots of stress against strain for commercial high molar mass PLLA and PLLA, blended with 2 wt % of **P1** and **P4**.

S6.3. DSC Data for PLLA Films with Different Weight Fractions of P1

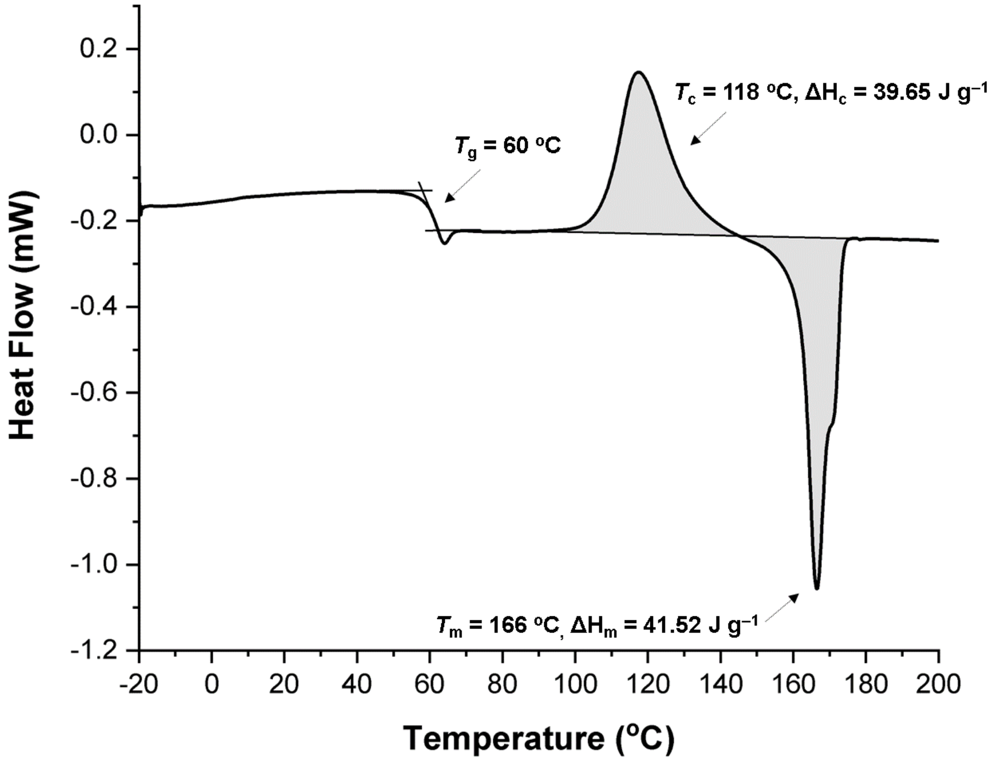

Figure S25. DSC thermogram of neat PLLA film.

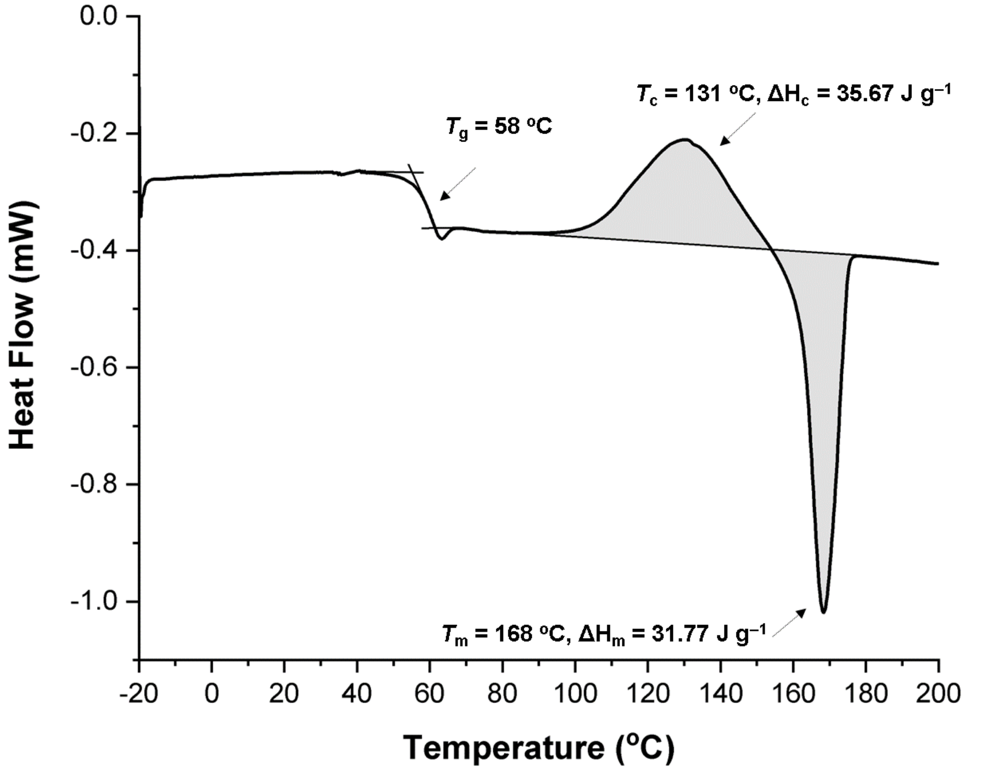

Figure S26. DSC thermogram of PLLA film blended with 2 wt % of **P1**.

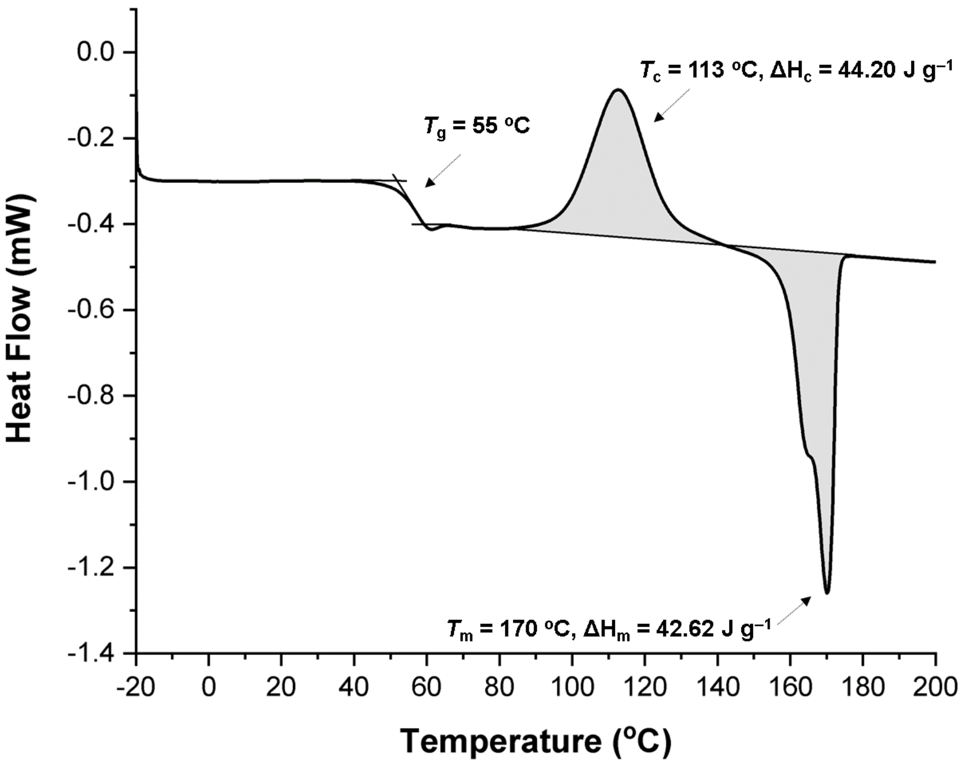

Figure S27. DSC thermogram of PLLA film blended with 5 wt % of **P1**.

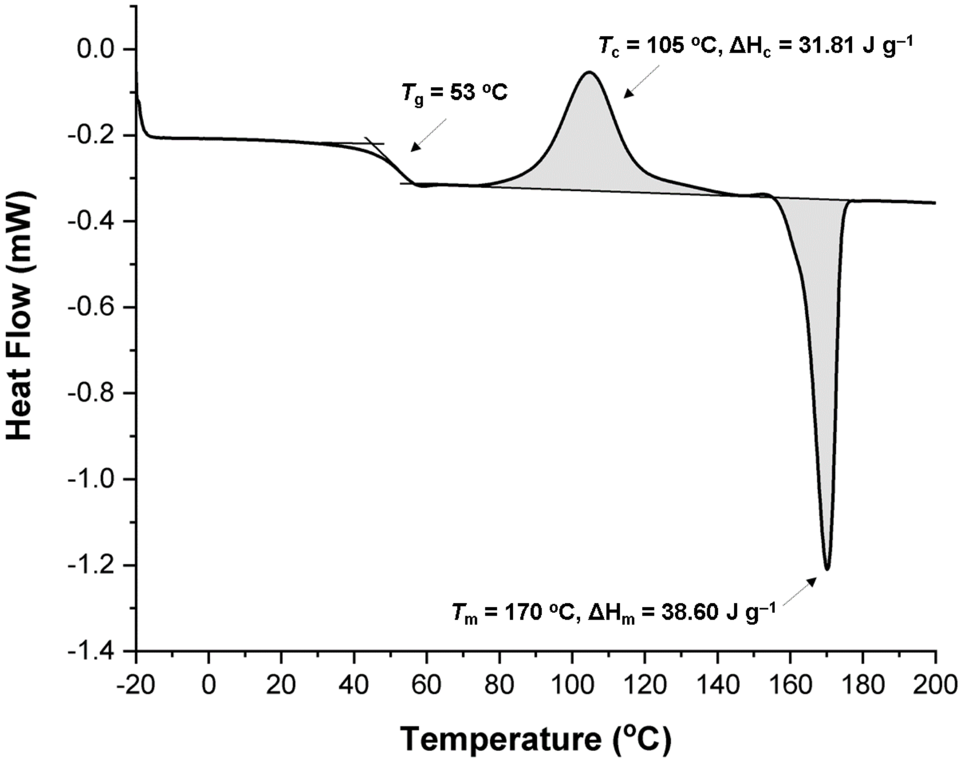

Figure S28. DSC thermogram of PLLA film blended with 10 wt % of **P1**.

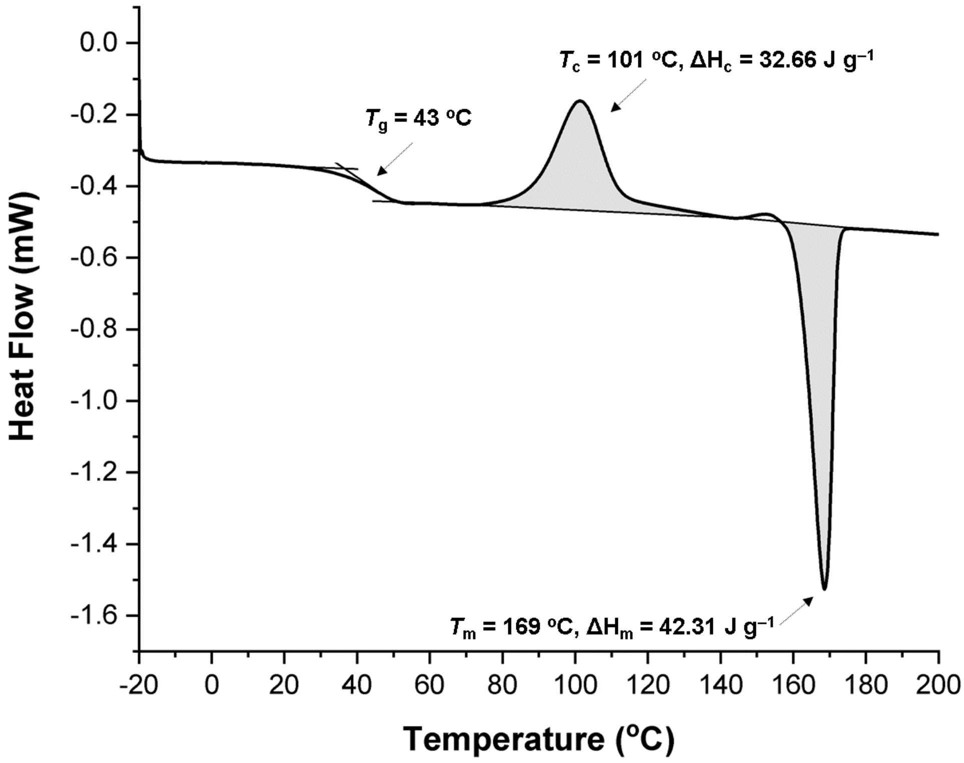

Figure S29. DSC thermogram of PLLA film blended with 20 wt % of **P1**.

S6.4. NMR Spectroscopic Data for Functionalized P2–P4

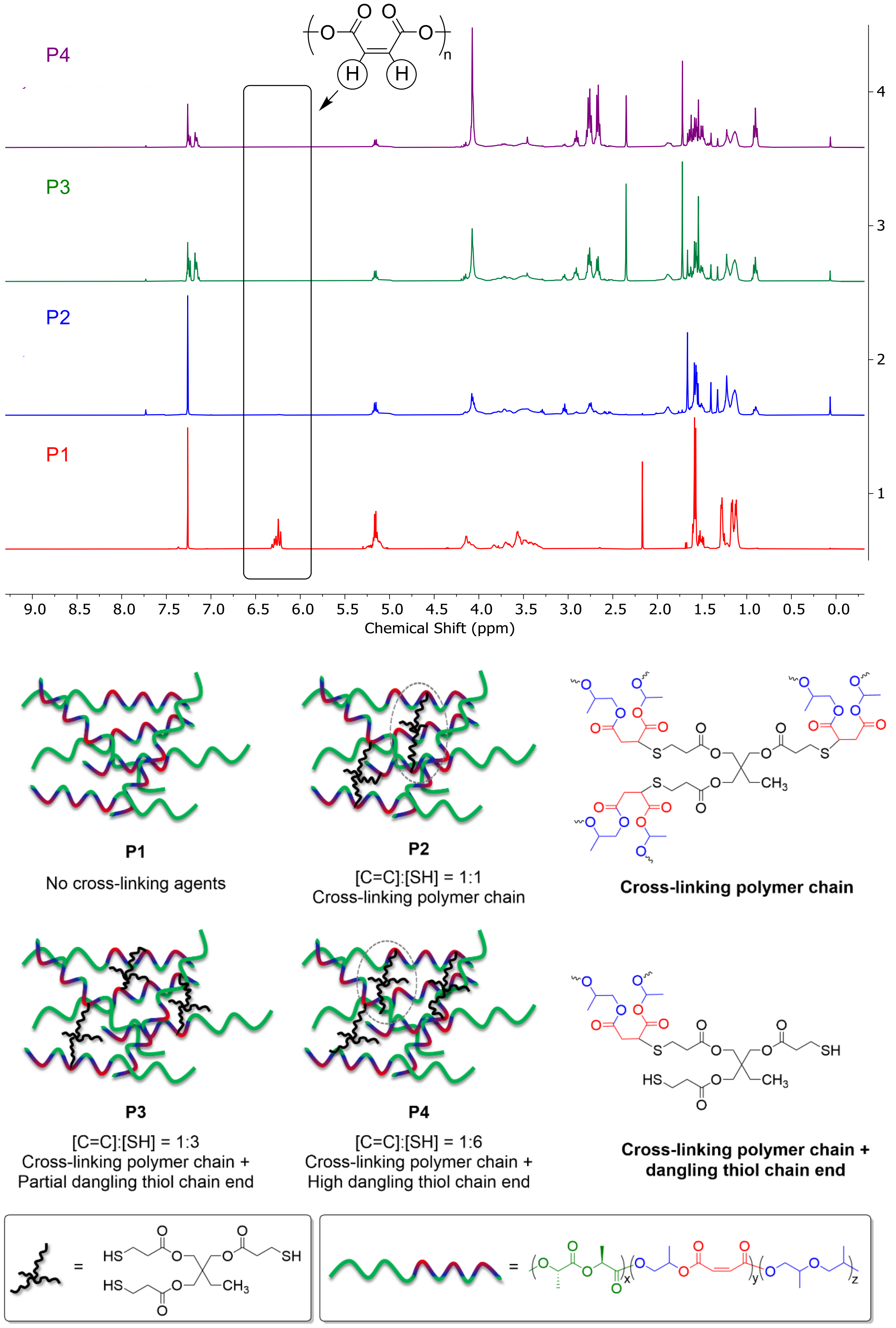

Figure S30. Stacked ^{1}H NMR spectra of **P1** (red), **P2** (blue), **P3** (green), and **P4** (violet) in $CDCl_3$ (Top) and cartoons represent proposed structures of **P1–P4** (bottom).

S6.5. DSC Data for Functionalized Polymers P2–P4

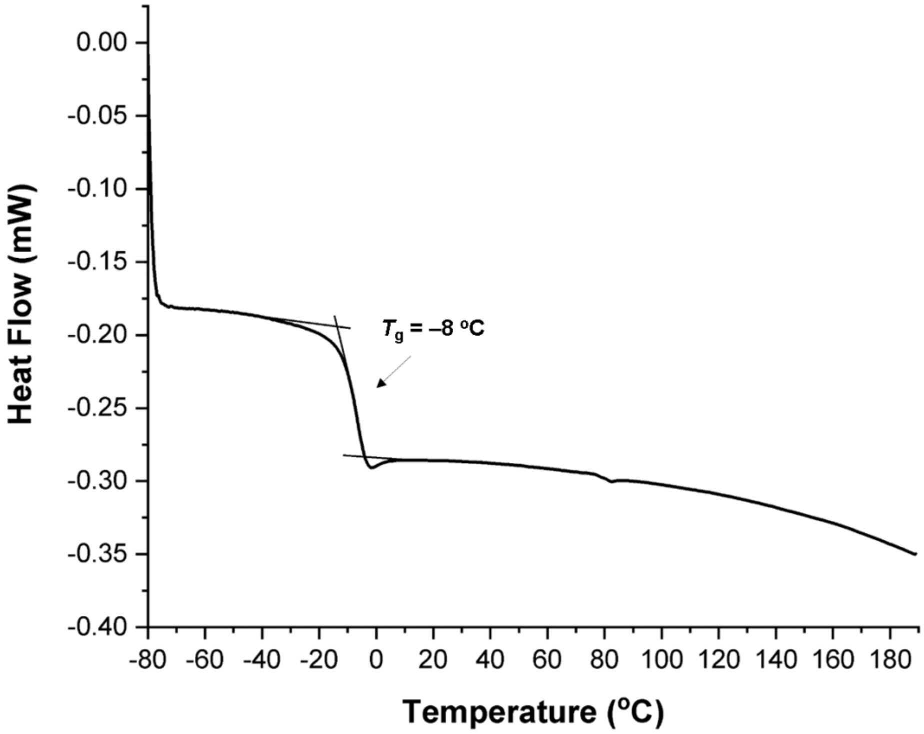

Figure S31. DSC thermogram of **P2**.

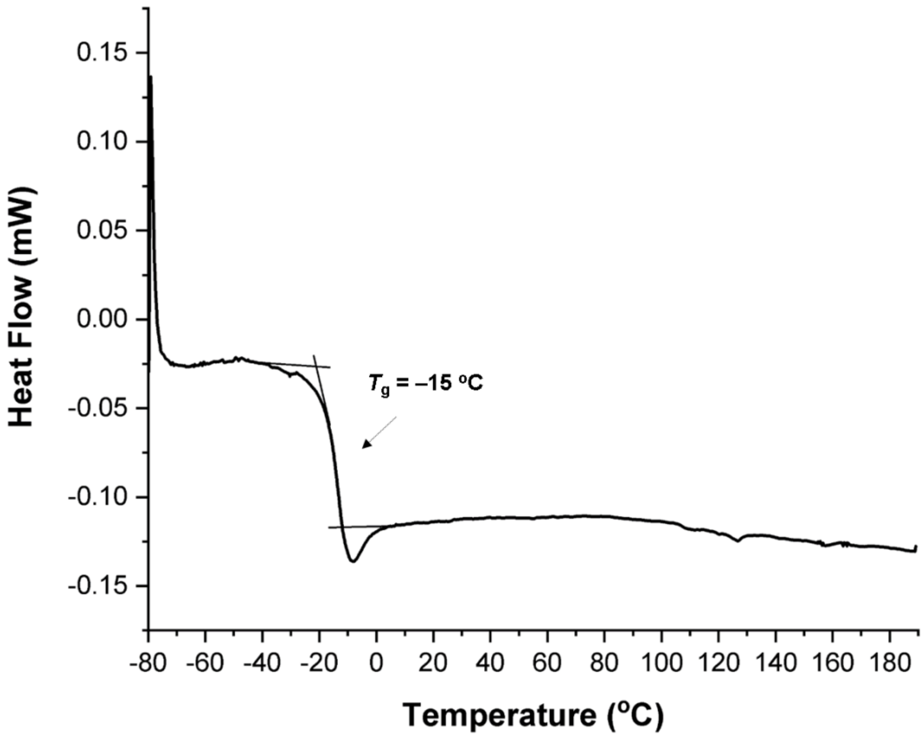

Figure S32. DSC thermogram of **P3**.

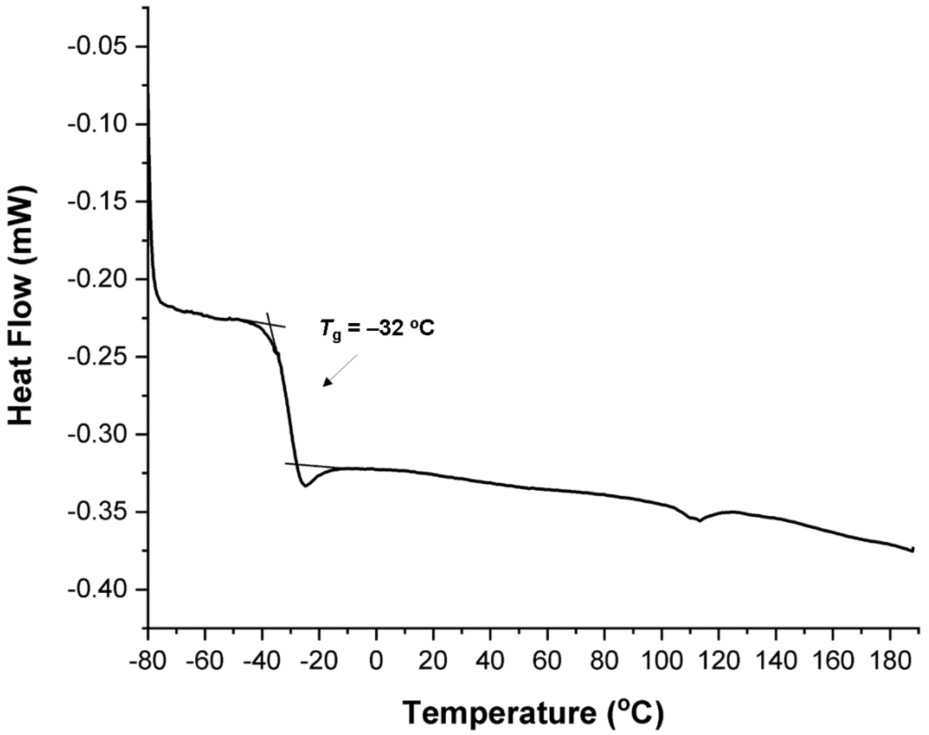

Figure S33. DSC thermogram of **P4**.

S6.6. TGA Data for Functionalized Polymers P2–P4

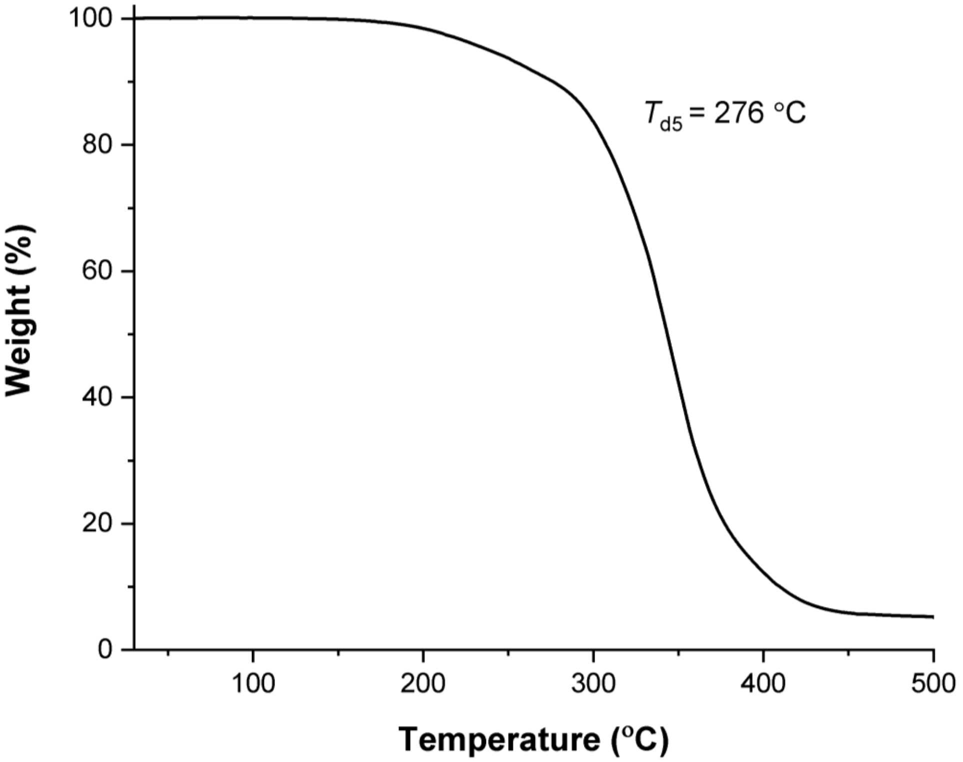

Figure S34. TGA thermogram of **P2**.

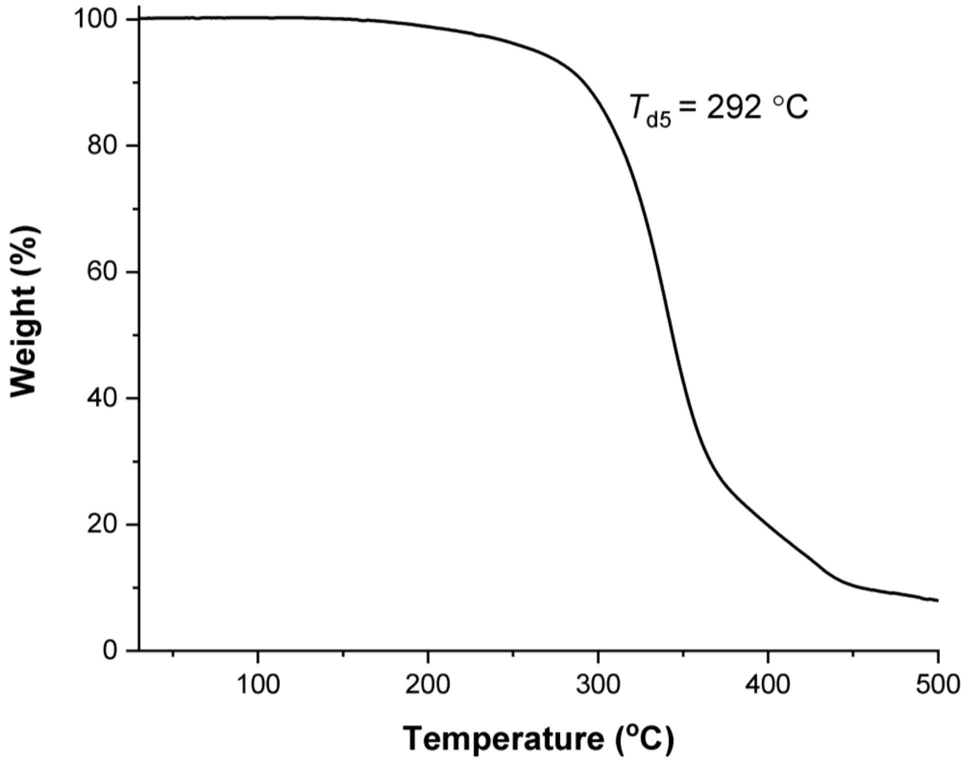

Figure S35. TGA thermogram of **P3**.

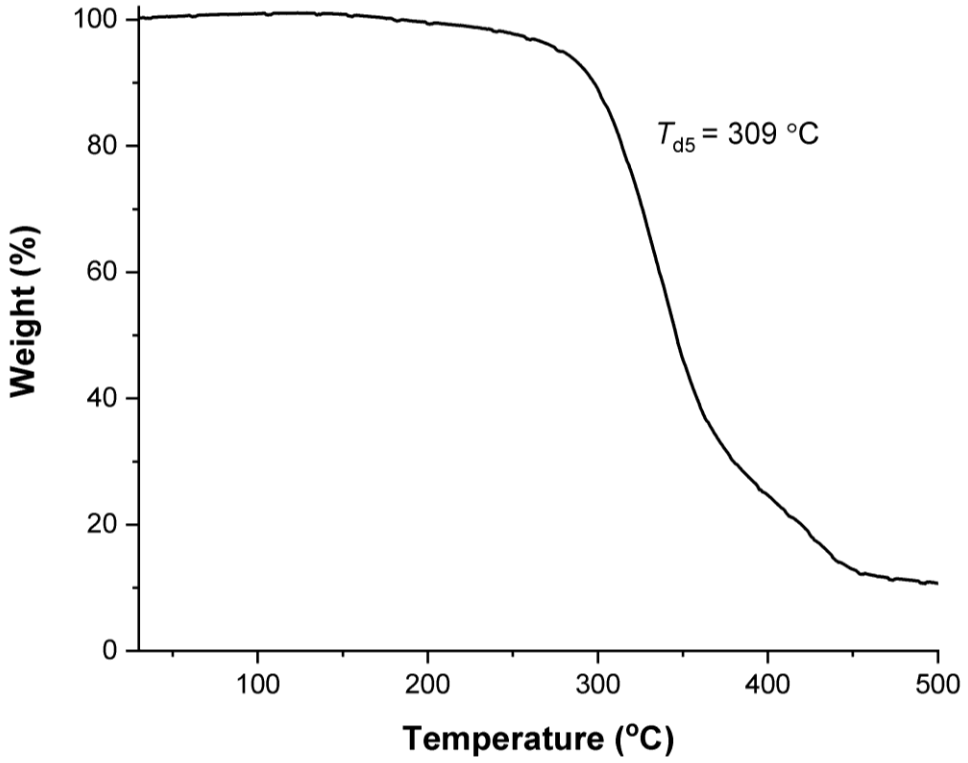

Figure S36. TGA thermogram of **P4.**

S6.7. Raman Spectra

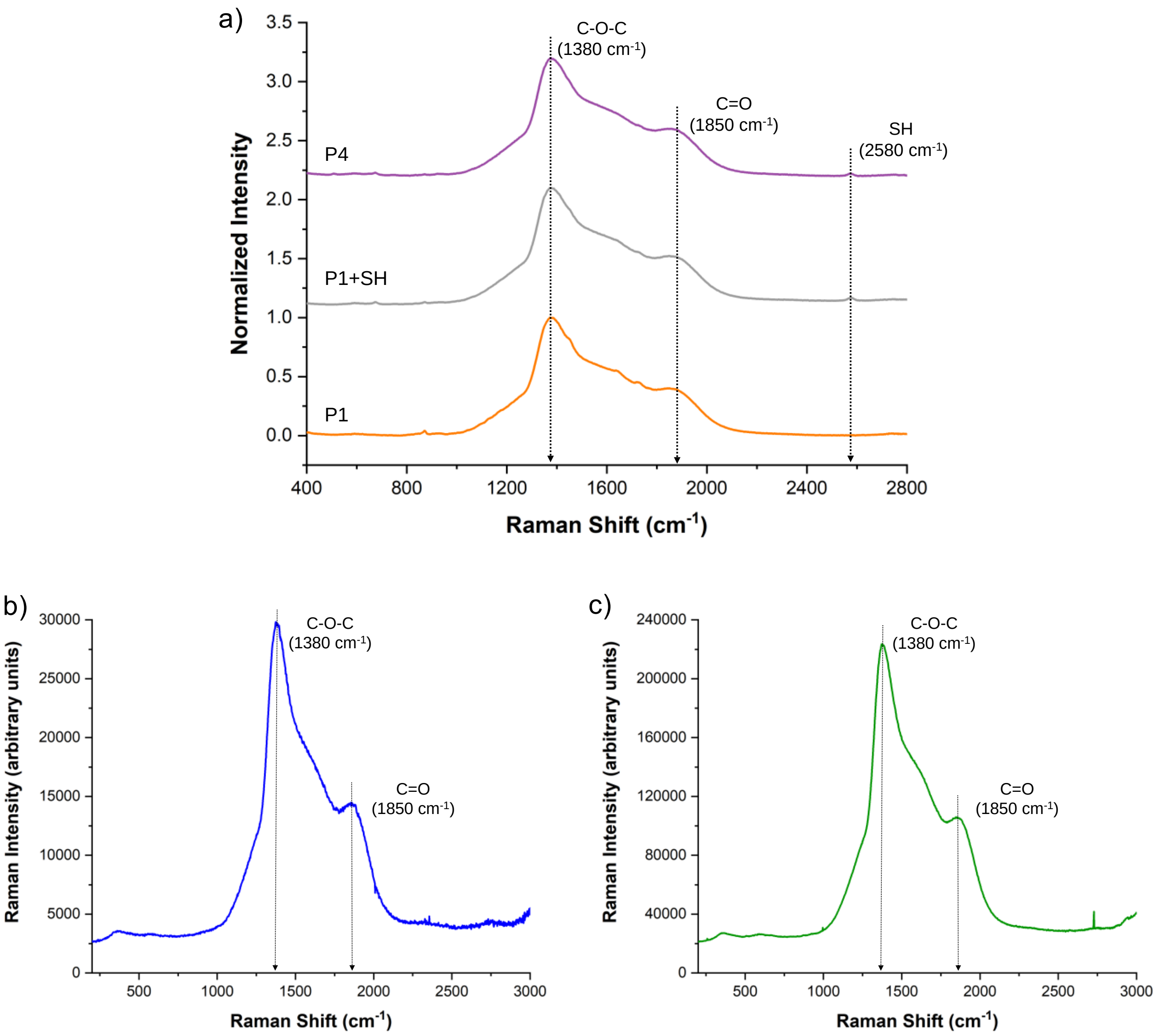

Figure S37. Raman spectra of the isolated polymers: a) Stacked spectra of **P1**, **P1**+ trimethylolpropane tris(3-mercaptopropionate), and **P4**, b) **P2**, and c) **P3**.

S6.8. DSC Data for PLLA Films with Two Weight Percent of P2–P4.

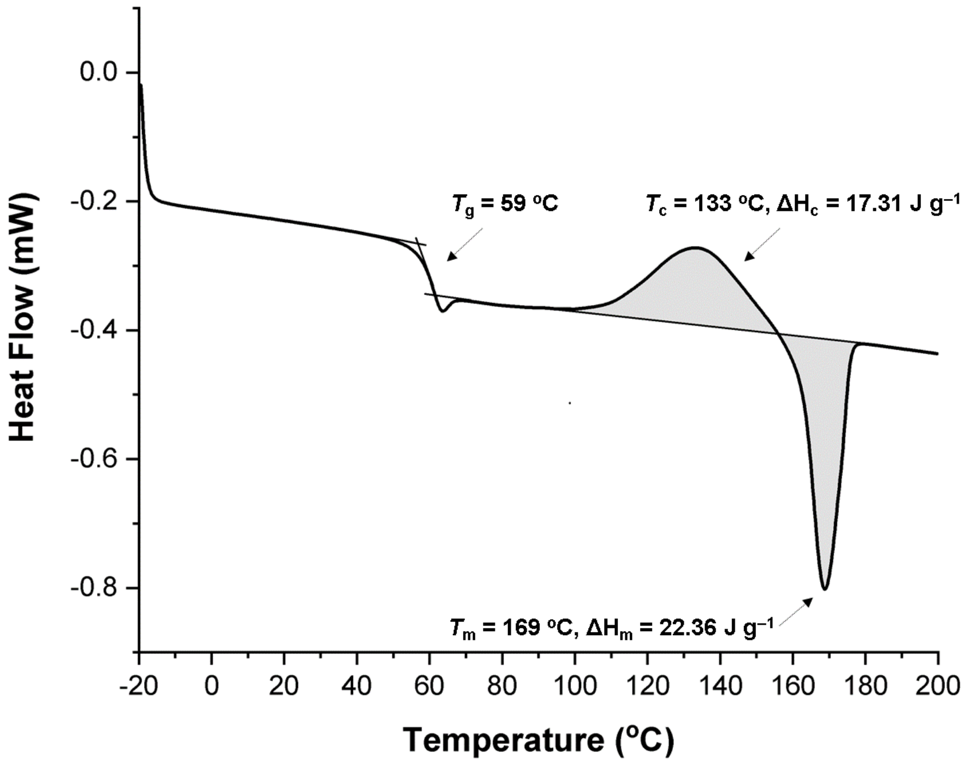

Figure S38. DSC thermogram of PLLA film blended with 2 wt % of **P2**.

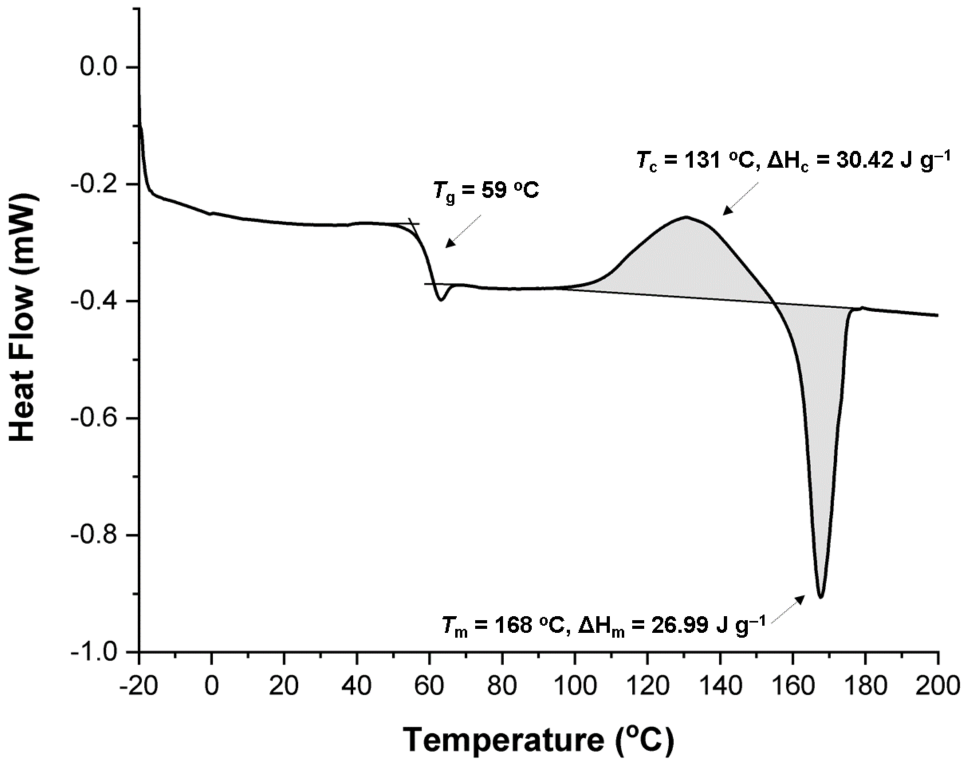

Figure S39. DSC thermogram of PLLA film blended with 2 wt % of **P3**.

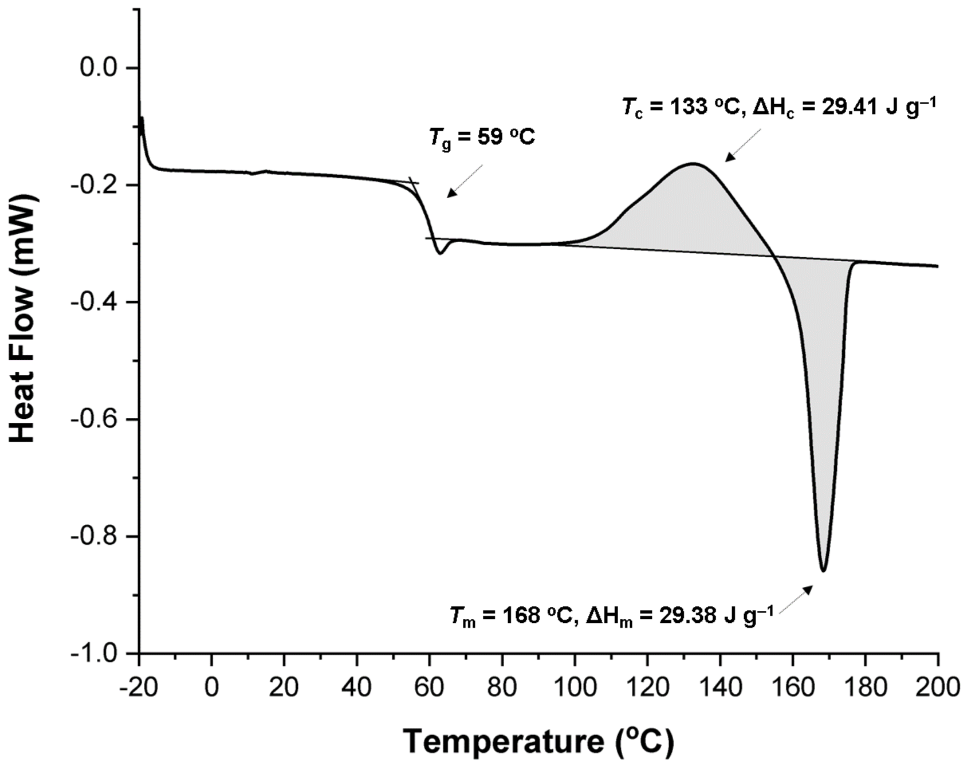

Figure S40. DSC thermogram of PLLA film blended with 2 wt % of **P4**.

S7. Comparative Data for Toughened PLLA Samples in the Literature

Table S6. Summary of additives used to toughen PLLA

Additive	PLLA:Additive	Young's Modulus (GPa)	Tensile Strength (MPa)	Yield Strength (MPa)	Elongation at Break (%)	Tensile Toughness (MJ m^{-3})	Ref
**P1**	98:2	2.20 ± 0.09 (no reduction)	56.5 ± 1.54 (11% lower)	66.2 ± 3.2 (6% lower)	11 ± 0.6 (1.2 times higher)	5.7 ± 0.49 (1.3 times higher)	This work
**P4**	98:2	1.99 ± 0.04 (11% lower)	49.6 ± 0.72 (22% lower)	58.4 ± 1.0 (18% lower)	36 ± 0.9 (4 times higher)	17.7 ± 0.21 (4 times higher)	This work
LLDPE [a]	80:20	1.30 ± 1.1 (45% lower)	24.3 ± 1.1 (61% lower)	NR	31 ± 18 (8 times higher)	7.7 ± 4.8 (5 times higher)	6
ABS [b]	70:30	1.61 ± 0.98 (10% lower)	40.6 ± 7.3 (25% lower)	63.4 ± 6 (2% lower)	151 ± 41 (18 times higher)	NR	7
PBA	92:8	1.49 (58% lower)	44.8 (34% lower)	NR	74.6 (16 times higher)	17.0 (8 times higher)	8
PCL	70:30	~1.70 (48% lower)	~40 (33% lower)	~40 (33% lower)	15 (5 times higher)	NR	9
PEO-*b*-PBO	95:5	2.0 ± 0.1 (15% lower)	NR	28 ± 4 (46% lower)	175 ± 24 (25 times higher)	26±4 (13 times higher)	10
PEO-*b*-PBO	98:2	2.8 ± 0.1 (7% lower)	NR	40 ± 3 (22% lower)	221± 46 (24 times higher)	58 ± 1.1 (24 times higher)	11
Tributyl-O-acetyl citrate	80:20	0.27 ± 0.02 (75% lower)	30.0 ± 1 (54% lower)	9 ± 1 (86% lower)	317 ± 4 (45 times higher)	NR	12
Bis(2-ethylhexyl) adipate	80:20	0.67 ± 0.12 (34% lower)	16.0 ± 2 (75% lower)	21 ± 1 (68% lower)	78 ± 33 (7 times higher)	NR	12
Glyceryl triacetate	80:20	0.01 ± 0.03 (100% lower)	24.0 ± 1 (63% lower)	NR	443 ± 13 (40 times higher)	NR	12
Tributyl citrate	80:20	NR	NR	7.1 (86% lower)	350 (50 times higher)	NR	13

Oligoester amide (M_n = 1 600 g mol^{-1}) [c]	85:15	NR	NR	~19 (50% lower)	200 (13 times higher)	NR	14

[a] 5 wt % of PLLA-*b*-polyethylene was used as a compatibilizer for polymer blend. [b] 3 wt % of random terpolymer of methyl methacrylate, glycidyl methyl acrylate and methyl methacrylate macromer was added as a compatibilizer. [c] Oligoesteramide was prepared from triethylene glycol diamine and pre-polymer which is the mixture of diethyl bishydroxymethyl malonate and adipyl dichloride.

S8. References

(1) Kowalski, A.; Duda, A.; Penczek, S., Polymerization of *L,L*-Lactide Initiated by Aluminum Isopropoxide Trimer or Tetramer. *Macromolecules* **1998,** *31*, 2114–2122.

(2) Robert, C.; Ohkawara, T.; Nozaki, K., Manganese-Corrole Complexes as Versatile Catalysts for the Ring-Opening Homo- and Co-Polymerization of Epoxide. *Chem. Eur. J.* **2014,** *20*, 4789–4795.

(3) DiCiccio, A. M.; Coates, G. W., Ring-Opening Copolymerization of Maleic Anhydride with Epoxides: A Chain-Growth Approach to Unsaturated Polyesters. *J. Am. Chem. Soc.* **2011,** *133*, 10724–10727.

(4) Takenouchi, S.; Takasu, A.; Inai, Y.; Hirabayashi, T., Effects of Geometrical Difference of Unsaturated Aliphatic Polyesters on Their Biodegradability II. Isomerization of Poly(maleic anhydride-co-propylene oxide) inthe Presence of Morpholine. *Polym. J.* **2002,** *34*, 36–42.

(5) Iannace, S.; Sorrentino, L.; Di Maio, E., 6 - Biodegradable biomedical foam scaffolds. In *Biomedical Foams for Tissue Engineering Applications*, Netti, P. A., Ed. Woodhead Publishing: 2014; pp 163–187.

(6) Anderson, K. S.; Hillmyer, M. A., The influence of block copolymer microstructure on the toughness of compatibilized polylactide/polyethylene blends. *Polymer* **2004,** *45*, 8809–8823.

(7) Dong, W.; He, M.; Wang, H.; Ren, F.; Zhang, J.; Zhao, X.; Li, Y., PLLA/ABS Blends Compatibilized by Reactive Comb Polymers: Double Tg Depression and Significantly Improved Toughness. *ACS Sustain. Chem. Eng.* **2015,** *3*, 2542–2550.

(8) Meng, B.; Deng, J.; Liu, Q.; Wu, Z.; Yang, W., Transparent and ductile poly(lactic acid)/poly(butyl acrylate) (PBA) blends: Structure and properties. *Eur. Polym. J.* **2012,** *48*, 127–135.

(9) Semba, T.; Kitagawa, K.; Ishiaku, U. S.; Hamada, H., The effect of crosslinking on the mechanical properties of polylactic acid/polycaprolactone blends. *J. Appl. Polym. Sci.* **2006,** *101*, 1816–1825.

(10) Li, T.; Zhang, J.; Schneiderman, D. K.; Francis, L. F.; Bates, F. S., Toughening Glassy Poly(lactide) with Block Copolymer Micelles. *ACS Macro Lett.* **2016,** *5*, 359–364.

(11) McCutcheon, C. J.; Zhao, B.; Jin, K.; Bates, F. S.; Ellison, C. J., Crazing Mechanism and Physical Aging of Poly(lactide) Toughened with Poly(ethylene oxide)-block-poly(butylene oxide) Diblock Copolymers. *Macromolecules* **2020,** *53*, 10163–10178.

(12) Murariu, M.; Da Silva Ferreira, A.; Alexandre, M.; Dubois, P., Polylactide (PLA) designed with desired end-use properties: 1. PLA compositions with low molecular weight ester-like plasticizers and related performances. *Polym. Adv. Tecnol.* **2008,** *19*, 636–646.

(13) Labrecque, L. V.; Kumar, R. A.; Davé, V.; Gross, R. A.; McCarthy, S. P., Citrate esters as plasticizers for poly(lactic acid). *J. Appl. Polym. Sci.* **1997,** *66*, 1507–1513.

(14) Ljungberg, N.; Wesslén, B., Preparation and Properties of Plasticized Poly(lactic acid) Films. *Biomacromolecules* **2005,** *6*, 1789–1796.
